# Simple Mathematical Models Do Not Accurately Predict Early SIV Dynamics

**DOI:** 10.3390/v7031189

**Published:** 2015-03-13

**Authors:** Cecilia Noecker, Krista Schaefer, Kelly Zaccheo, Yiding Yang, Judy Day, Vitaly V. Ganusov

**Affiliations:** 1 National institute for Mathematical and Biological synthesis (NIMBioS), Knoxville, TN 37996, USA; E-Mails: cecilia.noecker@gmail.com (C.N.); krista.schaefer@valpo.edu (K.S.); k.zaccheo@yahoo.com (K.Z.); judyday@utk.edu (J.D.); 2 Department of Genome Sciences, University of Washington, Seattle, WA 98195, USA; 3 School of Public Health, University of Illinois at Chicago, Chicago, IL 60612, USA; 4 School of Biomedical Engineering, Drexel University, Philadelphia, PA 19104, USA; 5 Department of Microbiology, University of Tennessee, Knoxville, TN 37996, USA; E-Mail: yidingyang@gmail.com; 6 Department of Mathematics, University of Tennessee, Knoxville, TN 37996, USA

**Keywords:** early SIV/HIV infection, mathematical model, eclipse phase, stochastic, Gillespie algorithm

## Abstract

Upon infection of a new host, human immunodeficiency virus (HIV) replicates in the mucosal tissues and is generally undetectable in circulation for 1–2 weeks post-infection. Several interventions against HIV including vaccines and antiretroviral prophylaxis target virus replication at this earliest stage of infection. Mathematical models have been used to understand how HIV spreads from mucosal tissues systemically and what impact vaccination and/or antiretroviral prophylaxis has on viral eradication. Because predictions of such models have been rarely compared to experimental data, it remains unclear which processes included in these models are critical for predicting early HIV dynamics. Here we modified the “standard” mathematical model of HIV infection to include two populations of infected cells: cells that are actively producing the virus and cells that are transitioning into virus production mode. We evaluated the effects of several poorly known parameters on infection outcomes in this model and compared model predictions to experimental data on infection of non-human primates with variable doses of simian immunodifficiency virus (SIV). First, we found that the mode of virus production by infected cells (budding *vs.* bursting) has a minimal impact on the early virus dynamics for a wide range of model parameters, as long as the parameters are constrained to provide the observed rate of SIV load increase in the blood of infected animals. Interestingly and in contrast with previous results, we found that the bursting mode of virus production generally results in a higher probability of viral extinction than the budding mode of virus production. Second, this mathematical model was not able to accurately describe the change in experimentally determined probability of host infection with increasing viral doses. Third and finally, the model was also unable to accurately explain the decline in the time to virus detection with increasing viral dose. These results suggest that, in order to appropriately model early HIV/SIV dynamics, additional factors must be considered in the model development. These may include variability in monkey susceptibility to infection, within-host competition between different viruses for target cells at the initial site of virus replication in the mucosa, innate immune response, and possibly the inclusion of several different tissue compartments. The sobering news is that while an increase in model complexity is needed to explain the available experimental data, testing and rejection of more complex models may require more quantitative data than is currently available.

## 1. Introduction

Acute infection with human immunodeficiency virus 1 (HIV-1 or simply HIV) consists of several well-defined phases. The majority of transmissions occur through vaginal or rectal intercourse, when the virus is able to cross the epithelial layer of the genital tract or rectal mucosa [1,2]. For approximately two weeks, the infection remains undetectable in the blood [3,4]. Viral replication during this period, called the eclipse phase, is thought to be a highly stochastic process [5]. Viral load in the blood reaches a peak at about 3–4 weeks post initial infection [3,4], which corresponds to the time when the HIV-specific CD8 T cell responses are detectable [1]. The rise of the HIV-specific CD8 T cell response correlates with a decline in viremia, and over time, a relatively constant viral load is established.

To better study early events of virus replication, a useful experimental model for HIV infection is simian immunodeficiency virus (SIV) infection of nonhuman primates of Asian origin [5,6,7,8,9]. In particular, seminal studies by the Haase group determined the sequence of events occurring at the mucosal sites following a large initial dose infection of macaques [10,11,12]. Other studies have examined how varying doses of the virus affect the probability of established infection and the time it takes for the virus to become detectable and reach a peak in peripheral blood in monkeys [13,14,15,16]. SIV infection of monkeys has already been a valuable experimental model for testing of HIV vaccines [17,18].

Many of the interventions to halt HIV epidemics, including vaccines and post-exposure antiviral treatment, target virus replication at a very early stage of infection. Vaccines inducing high levels of neutralizing antibodies have been shown to reduce the probability of infection with SIV in vaccinated monkeys [17], while vaccines inducing high levels of virus-specific CD8 T cells either control virus growth within the first few weeks post infection or fail [18]. Post exposure prophylaxis (PEP), another therapy, aims to prevent infection using antiretroviral drugs following viral exposure [19]. While there have been recent successes with the development of novel vaccine candidates and optimization of antiviral treatment to prevent infection [17,18,20], more research is likely needed to improve the vaccination and treatment schedules. Mathematical models focused on unraveling early virus dynamics can be useful in helping to improve strategies of viral control. However, because very limited quantitative information is available about HIV/SIV replication in mucosal tissues, development of an appropriate mathematical model of early HIV/SIV dynamics is not a trivial exercise.

To date, mathematical models of different levels of complexity have been proposed to study early HIV/SIV dynamics [21,22,23,24]. One set of mathematical models is based on the so-called “standard” model for HIV dynamics developed in early 1990s to describe viral dynamics in chronic infection following antiretroviral treatment [25,26,27]. The standard model assumes a well-mixed single compartment for HIV/SIV infection and includes the dynamics of uninfected target (CD4 T) cells, virus-infected cells that produce viral particles, and cell-free viral particles. This model has recently been used to investigate the role of virus production mode by infected cells on HIV dynamics in the first several weeks post-infection [22,23]. In the model, virus-infected cells can release virus particles (virions) either (1) on a continuous basis, immediately after a cell is infected lasting until cell death (continuous or budding production); or (2) all at once, either at the time of cell death or shortly before it (bursting production). Although this model is built on biologically reasonable assumptions and describes well the virus dynamics during antiviral therapy in chronically-infected patients [26,28], but it remains unclear whether the model is also quantitatively consistent with experimental data on HIV/SIV dynamics during the first 2 weeks post infection. Predictions coming from simple mathematical models may strongly depend on the particular choice of parameter values used for simulations, many of which are not well known, as well as on the overall structure of the models [29]. Furthermore, more complex models, including several different compartments such as mucosal and lymphoid tissues have been proposed and it remains unknown whether such increased model complexity is needed to explain quantitative experimental data [24].

In this work, we expand the standard HIV/SIV mathematical model, use it to investigate whether the virus production mode has a strong impact on the *early* virus dynamics, and evaluate whether it can describe available experimental data (e.g., data in Liu *et al.* [16]). In our approach, we incorporate an exponentially distributed delay between infection of a cell by a virus and production of virus particles by the infected cells suggested by previous publications [30,31]. Specifically, our model explicitly includes a cellular eclipse phase in the viral life cycle. By varying the relative average duration of the eclipse phase, the mode of virus production can vary continuously from budding to bursting. Note that this cellular eclipse phase between the infection of a cell and virus production by a cell is different from the period between infection and detection of the virus in the blood, which is also commonly referred to as an eclipse phase [1].

The paper is structured as follows. We first describe the model and properties of its asymptotic behavior, and then discuss constraints on the model parameters imposed by experimental data (Section 2.1). We then proceed with numerical simulations of the model dynamics, both deterministic and stochastic (Section 2.2). Finally, we discuss the predictions of the model on the change in the probability of established infection and the time to virus detection with increasing viral doses and compare model predictions to previously published experimental data (Section 2.3). We conclude the paper by discussing implications of our results for predicting early HIV/SIV dynamics.

## 2. Results

### 2.1. Extended Standard Mathematical Model for HIV/SIV Dynamics

#### 2.1.1. Mathematical Model

Several previous studies have described HIV dynamics during the first weeks post infection using mathematical models [21,22,23,24,30]. Some of these studies used the so-called “standard” model for HIV dynamics [27]. To run stochastic simulations of virus dynamics with this model when the number of viruses and infected cells is small requires making an assumption of how infected cells produce the virus. Two modes of virus production were thus postulated: continuous production (budding) or bursting production (virus production following cell death) [22,23]. To determine whether a continual change in the mode of virus production may impact the virus dynamics, we use an extended version of the standard model which includes an eclipse phase in the virus replication cycle like that found in previous publications [30,31,32].

**Figure 1:**
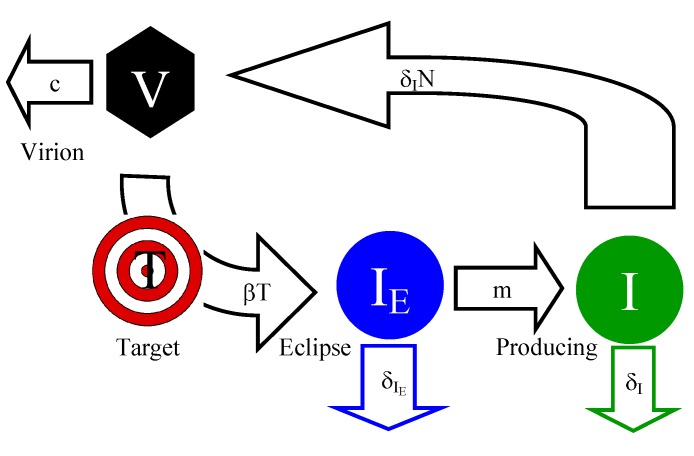
Interaction diagram of the extended standard mathematical model of the early dynamics of HIV/SIV. Viruses, *V* , infect target cells, *T* at a rate *β* and are cleared at a rate *c*. The target cell enters an eclipse phase, *I*_*E*_, where it does not actively release any virions. Cells in the eclipse phase die at a rate *δ*_*I_E_*_. From the eclipse phase, the cell transitions to the state of a productively-infected cell, *I*, at a rate *m*. Productively infected cells die at a rate *δ_I_* or release infectious virions at a rate *Nδ_I_*; *N* is the average number of infectious virions produced by an infected cell.

In this “extended” standard mathematical model we allow for two types of virus-infected cells: cells in the eclipse phase which are not making the virus, *I*_*E*_, and cells that are actively producing the virus, *I* (Figure 1). Cells in the eclipse phase transition to the state of virus production at a rate, *m*, and both types of cells die at rates $\delta_{I_{E}}$ and *δ_I_*, respectively. Cells in the eclipse phase may die because they could be recognized as infected by mediators of innate immunity (e.g., macrophages or NK cells), due to the activation of DNA-dependent protein kinase during integration of viral DNA into host chromosome or due to accumulation of DNA intermediates in the cell’s cytoplasm [33,34]. In fact, it has been argued that at least *in vitro* most HIV-infected cells die before virus production begins [33]. Virus-producing cells make infectious viruses, *V*, at a rate *Nδ_I_* where *N* is the average number of infectious virions released by an infected cell per its lifetime (burst size). It is generally accepted that a majority of virions produced by infected cells are non-infectious [26,35,36]. Since these viruses are not contributing to the infection of new cells, non-infectious viruses are not tracked in this model (e.g., see [22]). Viruses are removed from the cell-free virus population by either an intrinsic clearance rate, *c*, or by infecting target cells, which occurs at a rate *βT*, where *T* is the (constant) number of available target (CD4 T) cells. The model is then given by the following system of ordinary differential equations:

(1) $$\frac{dI_{E}}{dt}=\beta TV-(m+\delta_{I_{E}})I_{E}$$

(2) $$\frac{dI}{dt}=mI_{E}-\delta_{I}I$$

(3) $$\frac{dV}{dt}=\delta_{I}NI-(\beta T+c)V$$

In this well-mixed model, our assumption that *T* is constant during the initial stages of the infection is reasonable because the virus population grows exponentially at early stages of infection [16,37,38,39].

#### 2.1.2. Relative Duration of the Eclipse Phase

To study the effect of the virus production mode on virus dynamics in our model, we calculate the relative duration of the eclipse phase in the total life-span of infected cells. The average time that cells spend in the eclipse phase and as virus-producing cells is 1/(*m* + *δ*_*I_E_*_) and 1/*δ_I _*, respectively. Therefore, the fraction of total time cells spend in the eclipse phase is

(4) $$T_{m}=\frac{\frac{1}{m+\delta_{I_{E}}}}{\frac{1}{m+\delta_{I_{E}}}+\frac{1}{\delta_{I}}}=\frac{\delta_{I}}{m+\delta_{I}+\delta_{I_{E}}}\;\;$$

Depending on the values of *m*, *δ_I_*, and *δ*_*I_E_*_, the relative duration of the cellular eclipse phase, *T_m_*, can be set to emulate a cellular eclipse phase of a short, intermediate, or long duration. Subsequently, when the average life-span of infected cells is fixed, this provides a method to continuously vary the mode of virus production from budding-like to bursting-like.

#### 2.1.3. Asymptotic Behavior of the Model

Since the model (see Equations (1)–(3)) is linear, it has only two outcomes: exponential growth or exponential decline. Exponential decline (virus extinction) is guaranteed if the basic reproductive number, *R*_0_, is less than one (*R*_0_ < 1). As we show in Supplement, in this model the basic reproductive number is given by

(5) $$R_{0}=\frac{\beta T(m(N-1)-\delta_{I_{E}})}{c(m+\delta_{I_{E}})}=\frac{\beta T}{c}\left(\frac{mN}{m+\delta_{I_{E}}}-1\right)$$

While *R*_0_ determines the long-term deterministic behavior of the model, it is not directly measurable. The basic reproductive number can be calculated from observable rates of virus growth and death assuming different modes of virus production by infected cells [40,41]. Therefore, to relate *R*_0_ to the parameters that can be directly observed *in vivo* we calculated the rate at which the virus population expands during the phase of exponential growth or decays if virus replication is blocked, for example, during the use of highly active antiretroviral therapy (HAART) shortly after infection.

We followed the method used by De Boer [30], making the assumption that the clearance rate of viruses is large enough (*c* ≫ *δ_I_*, *c* ≫ *m*, *c* ≫ *δ*_*I_E_*_) that *V* is essentially a function of *I*:

(6) $$V\left(t\right)\approx \frac{N\delta_{I}}{c}I(t)$$

Then, the dynamics of the ratio *B* = *I/I_E_* of the number of virus-producing cells to the number of cells in the eclipse phase are

(7) $$\frac{dB}{dt}=m+(\delta_{I_{E}}+m-\delta_{I})B-\left(\frac{\beta TN\delta_{I}}{c+\beta T}\right)B^{2}$$

During the exponential phase of growth, the ratio *B* approaches a constant, so $\frac{dB}{dt}=0$, and the value of the ratio is found by solving the following equation:

(8) $$m+(\delta_{I_{E}}+m-\delta_{I})B-\frac{\beta TN\delta_{I}}{c+\beta T}B^{2}=0$$

When *δ_I_* + *m* − *δ*_*I_E_*_ > 0, there is one positive root in Equation (8), *B* = *B^∗^*. The rate of exponential increase of the viral population, *r*, is then given by

(9) $$\;r=\frac{\left(\frac{\beta TN\delta_{I}}{c+\beta T}-\delta_{I}\right)B^{*}-\delta_{I_{E}}}{1+B^{*}}$$

From Equation (9), we can express *δ_I_* as the function of all other parameters

(10) $$\delta_{I}=\frac{\delta_{I_{E}}+(1+B^{*})r}{B^{*}\left(\frac{\beta TN}{c+\beta T}-1\right)}$$

If the host is treated with antiretroviral drugs, we expect that the viral load will decay exponentially [42]. Straightforward calculations show that when new infections are blocked with the use of 100% efficacious reverse transcriptase inhibitor, making *βT* = 0, the number of virus-producing cells, *I*, declines exponentially as seen in the following equation:

(11) $$I(t)=I(t_{0})e^{-\delta_{I}(t-t_{0})}+\frac{mI_{E}(t_{0})}{\delta_{I}-(m+\delta_{I_{E}})}(e^{-(m+\delta_{I_{E}})(t-t_{0})}-e^{-\delta_{I}(t-t_{0})})$$

where *I*(*t*_0_) and *I_E_*(*t*_0_) are the numbers of virus-producing cells and cells in the eclipse phase, respectively, at the time of the start of the therapy *t*_0_. Due to a short half-life of virions, the dynamics of the virus is proportional to that in Equation (11). Thus, in this model, the asymptotic virus decline during HAART is governed by the smaller of the two values, *δ_I_* or *m* + *δ*_*I_E_*_.

#### 2.1.4. Probability of Extinction

In the case when *R*_0_ < 1, virus extinction is guaranteed. However, if *R*_0_ > 1, virus extinction may occur with some probability depending on the parameters and whether infections start with free virus or virus-infected cells. In the case when the infection is started with one virion, the majority of extinctions will occur because the virion is cleared (*c*) before infecting a target cell (*βT*); the probability of this happening is simply $\frac{c}{c+\beta T}$. If infection of a cell does occur, then extinction will occur if the cell in the eclipse phase dies (*δ*_*I_E_*_) before maturing (*m*) into virus-producing cell; the probability of this happening is $\frac{\delta_{I_{E}}}{\delta_{I_{E}}+m}\;$. Finally, the probability of extinction depends on the number of infectious viruses produced by a virus-producing cell during its lifespan. Taken together, the probability that the infection becomes extinct when starting with a single virion (*V*_0_ = 1) is given by

(12) $$q=\frac{c}{c+\beta T}+\frac{1}{N}+\frac{\beta T}{c+\beta T}\frac{\delta_{I_{E}}}{m+\delta_{I_{E}}}$$

(see Supplement for derivation). Furthermore, assuming that all viruses act independently as the model (given in Equations (1)–(3)) implies, the probability that an infection is established given that *V*_0_ ≥ 1 virions are initially present is

(13) $$p_{\text{inf}}=1-q^{V_{0}}=1-e^{-\lambda D}$$

where the initial number of infectious virions is dependent on the initial viral dose *D*, *V*_0_ = *αD*, *λ* = −*α* ln *q* and *q* is given in Equation (12). This model is identical to “single-hit” models for exposure of tissues to radiation or infection of hosts with a given dose of a pathogen [43,44,45,46]. As we show below, this model is unable to accurately describe the data of Liu *et al.* [16].

We considered several extensions of this simple “single-hit” model. The first alternative model which we call a “power-law” model assumes that the change in the probability of infection with increasing viral dose is given by the relationship

(14) $$p_{\text{inf}}=1-e^{-\lambda D^{n}}$$

where we assume that infections by different virions do not occur independently, such that interactions between individual viruses are either antagonistic (*n* < 1) or synergetic (*n* > 1). Also, this model may be appropriate if there is a nonlinear relationship between the number of viruses, initiating the infection, and the initial dose, *V*_0_ = *αD^n^*.

The second alternative model, the “competition” model, assumes that the probability of established infection declines with the initial number of viruses *i*, such as *p_i_* = *p*_0_e^−^*^λi^*. In this model, a higher number of viruses leads to a lower probability of established infection, which indirectly implies antagonistic interactions between different viruses. The normalized probability of infection of the animal given initial number of viruses *V*_0_ = *αD* is then

(15) $$p_{\text{inf}}=\frac{e^{-\lambda }(1-e^{-\lambda \alpha D})}{1-e^{-\lambda (\alpha D+1)}}$$

It is important to note that in this model the probability of established infection does not approach 1 as *D* → *∞*, which makes it different from other models presented here. It may therefore be able to describe the resistance of some animals to repeated small doses of SIV as was illustrated recently [47]. We would like also to note that we could not find direct experimental evidence of synergetic or antagonistic interactions between different viruses, and thus, these interactions remain highly theoretical constructs.

Finally, the third alternative model, the “gamma” model, assumes that there is an intrinsic difference in susceptibility of animals to infection [45,46,48]. Susceptibility to infection in the basic model (Equation (13)) is given by the parameter *λ*. Thus, if the distribution of susceptibilities of different animals is given by a distribution *f* (*λ*)d*λ*, then probability of establishing infection is

(16) $$p_{\text{inf}}=1-\int f\left(\lambda \right)e^{-\lambda d}d\lambda$$

If *f* (*λ*) is given by the gamma distribution with the mean *λ* and variance *σ*^2^, $f\left(\lambda \right)=\frac{\lambda }{\sigma^{2}}\frac{{\left(\lambda \bar{\lambda }/\sigma^{2}\right)}^{{\bar{\lambda }}^{2}/\sigma^{2}-1}}{({\bar{\lambda }}^{2}/\sigma^{2}-1)!}e^{-\lambda \bar{\lambda }/\sigma^{2}}$, then the probability of infection is given by

(17) $$p_{\text{inf}}=1-{\left(\frac{\bar{\lambda }}{\bar{\lambda }+\sigma^{2}D}\right)}^{{\bar{\lambda }}^{2}/\sigma^{2}}$$

Gamma distribution for *λ* is chosen because it can be symmetric and nonsymmetric and allows for analytical expression of *p*_inf_.

#### 2.1.5. Parameter Estimations

There are no direct *in vivo* measurements of kinetic parameters determining HIV/SIV dynamics during the first several days post infection, before the virus is detectable in the blood. However, some estimates for these parameters can be made if we assume that the standard mathematical model (or its extension) is appropriate for description of the virus dynamics before the peak of viremia.

*Viral clearance rate*, *c*. Given the available measurements, it is natural to assume that the rate of clearance of viral particles is much higher than the death rate of virus-infected cells, even though these measurements were done in chronically infected animals [49].  Recent work also suggests that viral clearance rates in tissues could be even higher than that measured in the blood [50]. Thus, in our simulations we assumed clearance rate to be high *c* ≥ 20 day^−^^1^ (Table 1), although the exact value does not strongly influence model predictions as long as *c* is large enough, *i.e.*, *c* ≫ *δ_I_*, *m*, *δ*_*I_E_*_. 

*Constraints on parameters related to virus decay rate during treatment*. The kinetics of viral decay during HAART, as predicted by the mathematical model, are given by Equation (11) and are asymptotically determined by the minimal of the two rates, *m* + *δ*_*I_E_* or *δ_I_*_. This decay rate is approximately 0.5 − 1.5 day^−^^1^ as estimated in many experimental studies (e.g., [51,52,53,54]), although only one study (as far as we are aware) looked at viral decay rates during treatment of acute infections [42]. Therefore, the minimal of the two rates, *m* + *δ_I_* or *δ_I_*, should be approximately equal to 0.5 − 1.5 day^−^^1^.

*Eclipse phase transition rate*, *m*. Some studies suggest that it takes 24 h for a cell to begin producing virus after it is initially infected [54,55], but smaller estimates have been proposed [56]. Thus, the rate at which a cell transitions out of the eclipse phase and into the phase when it is actively producing virions, *m*, can be given the following range of values *m* = 0.5 − 2 day^−^^1^.

**Table 1:** An example of parameter values for the mathematical model used in several deterministic and stochastic simulations. Even though parameters were chosen to guarantee a net rate of increase of the viral load of *r* = 1.5 day^−^^1^ [16,39], the observed rates of expansion, *r_o_*, were lower due to a finite value for the clearance rate of the virus, *c*. The relative duration of the eclipse phase, *T_m_*, is given by Equation (4). Higher values of *T_m_* imply a long eclipse phase with burst-like virus production, and smaller values imply a short eclipse phase with budding-like virus production. Note that *m* = 5 day^−^^1^ and *m* = 0.7 day^−^^1^ are on the extremes of short and long average times of the duration of the eclipse phase: 5 and 34 h, respectively.

Parameter, Units	Virus Production Mode			Parameter Description	References	
	Continuous	Intermediate	Burst			
*δ*_*I_E_*_, day^−^^1^	0.5	0.5	0.5	death rate of cells in the eclipse phase		unknown
*m*, day^−^^1^	5.0	1.5	0.7	eclipse phase transition rate		[54,55]
*δ_I_*, day^−^^1^	0.583	1.313	5.06	death rate of virus-producing cells		unknown
min(*m* + *δ*_*I_E_*_, *δ_I_*)	0.583	1.313	1.2	virus decay rate during HAART		[51,52,53,54,58]
*c*, day^−^^1^	20	20	20	virion clearance rate		[49,50]
*N*	10	10	10	infectious virion burst size		unknown
*βT*, day^−^^1^	20	20	20	rate of infection		unknown
*T*_*m*_	0.10	0.40	0.81	relative duration of the eclipse phase		unknown
*r_o_*, day^−^^1^	1.443	1.445	1.433	observed net viral growth rate		[16,39]

*Death rate of eclipse phase cells*, *δ*_*I_E_*_. Previous models assumed that cells in the eclipse phase have a death rate that is zero or very small [24,30]. However, cells in the mucosal tissues may have an intrinsically high rate of removal due to their proximity to gut microbiota, the non-optimal (nonlymphoid) host tissue for survival, or recognition of virus-infected cells by tissue phagocytes. Recently, it has been observed that *in vitro* cell cultures infected with HIV die prior to reproducing the virus [33]. The proposed mechanism for this high death rate is the activation of cellular apoptotic mechanisms, triggered by the double strand breaks in DNA preceding integration of viral DNA into the chromosome. Alternatively, the induction of pyroptosis due to accumulation of DNA intermediates can also lead to self-induced death of HIV-infected cells prior to virus production [34]. Although it is not known whether such processes occur *in vivo*, it is possible that most of cell death occurring during virus replication occurs during the eclipse phase, prior to production of virions. In most of our simulations we assume that cells in the eclipse phase survive for about 2 days (*δ*_*I_E_*_ = 0.5 day^−^^1^, Table 1), but also check robustness of our results when *δ*_*I_E_*_ has lower values. In general, the value of *δ*_*I_E_*_ is not known.

*Relationship between the initial viral dose* *V*_0_ *and the average number of infectious viruses produced per infected cell* *N*. Although many studies report a particular dose used to infect animals with SIV, it is not known how many viruses actually penetrate the tissue and initiate the infection [13,14,15,16]. However, the fraction of animals that do get infected when exposed to a certain viral dose is generally known; therefore, for a given experiment, we can know *p*_inf_ (Equation (13)). To obtain further insights into how *N* and *V*_0_ may be related we first analyze a simplification of our model when the duration of the eclipse phase is small, *i.e.*, *m* → *∞*. At this limit the extended model approaches the standard model. Since lim*_m__→∞_* *B* = *∞* in Equation (9), by using basic algebraic manipulations and the formulas for Equations (9) and (13) we find the desired relationship

(18) $$V_{0}=\frac{\ln(1-p_{\text{inf}})}{\ln(1-\frac{r}{N\delta_{I}})}\approx \frac{N\delta_{I}a_{0}}{r}$$

where *a*_0_ = − ln(1 − *p*_inf_) and the approximation is found when *N* ≫ 1. This result suggests that for a fixed probability of infection, increasing the average number of infectious virions produced by an infected cell, *N* , increases the initial dose of the virus needed to produce the observed *p*_inf_. This relationship can alternatively be viewed as the virus burst size needed to explain the observed virus dynamics for a given initial virus density. For example, in the study by Liu *et al.* [16], 2 out of 6 macaques were infected with SIV at the lowest dose of 10^6^ viral particles and only a single transmitted/founder virus initiated the infection. Assuming that *V*_0_ = 1, *r* = 1.5 day^−^^1^ and *δ_I_* = 1 day^−^^1^ [16,39,51,52,53,54], the burst size must be around

$$N=\frac{1.5}{2/6\times 1}\approx 4.5\text{ infectious viruses}$$

in order to achieve a relatively high probability of infection *p*_inf_ = 0.33 and a rapid net rate of viral growth, *r*. This is surprisingly low given that it has been estimated that an SIV-infected cell produces around 5 × 10^4^ viral particles [57]. Several studies have suggested that SIV/HIV infectivity is relatively low and only 1 in 1000 viral particles are infectious [26,35]; however, a more recent study argued that the fraction of infectious viruses could be much higher [36]. We found in our simulations that if we adjust *V*_0_ and the level at which the virus population becomes detectable to match experimental data of Liu *et al.* [16], our modeling results are not very sensitive to the actual value of *N* (Figure S2 in Supplement).

Using the standard model (*i.e.*, when *m* → *∞* in our model) one can also calculate the level of virus infectivity, *βT*, that is required to generate the observed kinetics of increase in viral load, *r*. In the standard model, the rate of viral load increase is simply

(19) $$r=\frac{\beta TN\delta_{I}}{c+\beta T}-\delta_{I}$$

and consequently, the virus infection rate is then

(20) $$\beta T\approx \frac{c}{N}\left(1+\frac{r}{\delta_{I}}\right)$$

where the approximation is valid at *N* ≫ 1 + *r/δ_I_*. Because the extended standard model (Equations (1)–(3)) has 2 additional parameters (*m* and *δ*_*I_E_*_), no simple analytical relationship exists between *V*_0_ and *N* in this model. Therefore, to run deterministic and stochastic simulations in the extended model we use the following algorithm. First, we choose values for a set of parameters, for example, *m*, *δ*_*I_E_*_, and *N* within biologically reasonable range (as discussed above). Then, we calculate the values of the remaining parameters (e.g., *βT* and *δ_I_*) given the constraints set by the calculations for *r*, min(*δ*_*I*_, *m* + *δ*_*I_E_*_), and *p*_inf_, and run simulations using the resulting parameters. This procedure is repeated for various sets of parameter values to determine the overall behavior of the model. An example of the parameter values used in some simulations is shown in Table 1.

*Viral* *detection level*. The total amount of the virus in the body at which the virus becomes detectable in the blood is not known. Traditional experimental detection methods allow detection of 50–200 copies of viral RNA per mL of blood, but how this is translated into the total amount of the virus in the body has not been defined (see also our calculations in the Discussion). To define the detection limit *V*_det_ we use experimental data from Liu *et al.* [16]. In these experiments, monkeys were exposed to variable doses of SIVmac251 intrarectally and the virus kinetics in infected animals were followed over time. The authors found that at the lowest viral dose (10^6^ viral particles) the virus became detectable in the blood on average 8.5 days post infection [16]. Therefore, for a given set of model parameters, *V*_det_ is determined empirically by running deterministic simulations of the model (Equations (1)–(3)) and defining *V*_det_ as the virus density at time *t* = 8.5 days since infection. Given that we have bounded the parameters of the model to satisfy the measured constraints (see Table 1 and Equation (18)), both *V*_det_ and *V*_0_ are directly proportional to the burst size, *N*, (Figure S2 in Supplement). As an example, for the parameters shown in Table 1 and for *V*_0_ = 1, the detection limit is *V*_det_ ≈ 10^4^ (Figure 2 in Supplement).

### 2.2. Simulating Virus Dynamics

#### 2.2.1. In Deterministic Simulations, the Mode of Virus Production by Infected Cells Does Not Strongly Impact the Time to Virus Detection

**Figure 2:**
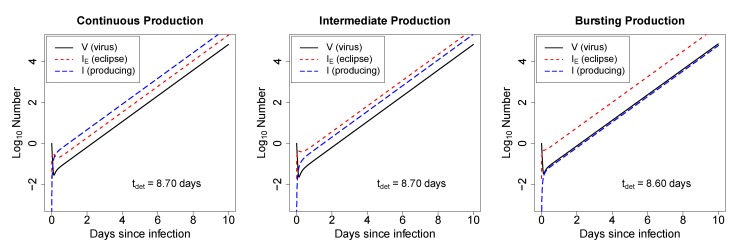
For biologically reasonable parameter values, the mode of virus production by infected cells has little impact on the time to virus detection in deterministic simulations. We solve the mathematical model (Equations (1)–(3)) numerically and plot predicted values for the infectious virus (*V*), infected cells in the eclipse phase (*I_E_*), and virus-producing cells (*I*). Time courses are shown for the model with continuous-mode virus production (short eclipse phase, left panel), intermediate mode virus production (eclipse phase takes approximately half of the life-span of infected cells, middle panel), and bursting mode of virus production (long eclipse phase, right panel). Parameters used are given in Table 1. All infections are started with *V*_0_ = 1. The time of virus detection, *t*_det_, is the time when the virus population reaches *V*_det_ = 10^4^ infectious viruses and is indicated on individual panels.

To explore the dynamics of our mathematical model (Equations (1)–(3)) we ran a set of deterministic simulations. When starting an infection with one virion, there is an initial decline in the number of virions before it grows exponentially (Figure 2); the rate of exponential decline is approximately given by virus clearance rate *c* (see Supplement for derivations). Although biologically unrealistic, the deterministic model allows for a fraction of one virus to exist, and thus can rebound from a number less than one. For this set of parameters, the number of infectious viruses present at any time is predicted to be lower than the number of cells in the eclipse phase or the number of virus-producing cells (Figure 2). This arises because our model tracks the dynamics of only infectious viruses and because in our simulations only *N* = 10 infectious viruses are produced on average by the virus-producing cells. Increasing the burst size results in simulations in which the amount of free virus exceeds the amount of virus-infected cells (Figure S2 in Supplement). Importantly, as expected from the standard model for HIV dynamics [27], the mode of virus production, either bursting or budding, has little impact on the time to virus detection in such deterministic simulations (Figure 2). Interestingly, if infections are started with 1 cell in the eclipse phase or with 1 virus-producing cell, the populations approach the phase of exponential growth more rapidly than when infection starts with a single virion (results not shown).

#### 2.2.2. In Stochastic Simulations, the Initial Viral Dose Impacts the Time to Virus Detection but the Mode of Virus Production Does Not

Due to finding that early infections likely start with a small number of founding viruses [15], we simulated the dynamics of our model using the Gillespie algorithm for small population sizes ([59], see Materials and Methods). Simulations illustrate that it takes several days for a stochastic model to reach the regime of exponential growth, and that stochastic runs can often achieve higher virus numbers than that predicted by the deterministic simulations (Figures S1 and S2 in Supplement).

**Figure 3:**
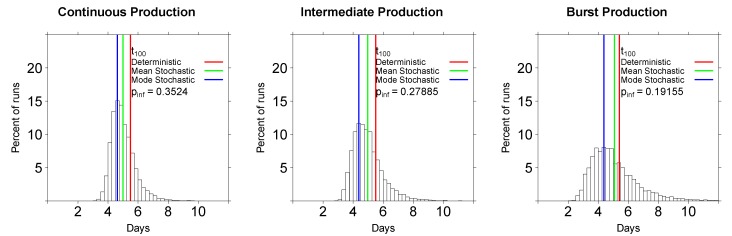
Distributions of times to 100 infectious viruses obtained using the Gillespie algorithm for the extended standard mathematical model of HIV/SIV dynamics (see Equations (1)–(3)). Simulations are for continuous/budding viral production mode (short eclipse phase, left panel), intermediate viral production mode (middle panel), and burst-like viral production mode (long eclipse phase, right panel). All infections start with one infectious virion, *V*_0_ = 1. We performed 20,000 simulations for every parameter set (Table 1). Note that the time to 100 infectious viruses is shorter than the average time to virus detection as observed in experiments of Liu *et al.* [16] (see also Figure 6).

Because simulating the virus dynamics to 10^4^ infectious viruses was prohibitively slow, we gained initial insights into the impact of parameter values on virus dynamics by calculating the time to 100 infectious viruses, *t*_100_. There was a wide distribution of times to 100 infectious viruses in our stochastic simulations (Figure 3). We characterized these distributions by calculating the mean and mode of the distribution of *t*_100_ for all simulations. Because the *t*_100_ distributions were not highly skewed, the mean and median time to detection were nearly identical (results not shown). A number of interesting results emerged from these simulations.

**Figure 4:**
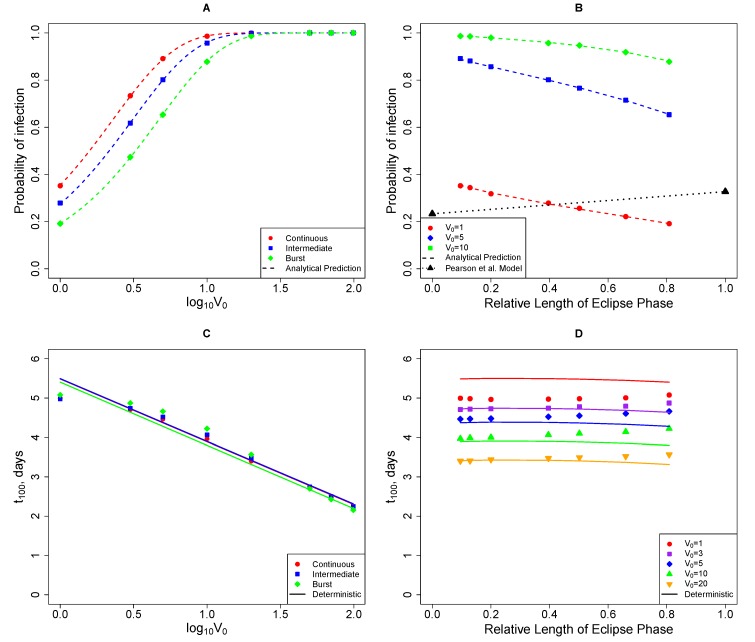
Changes in the probability of established infection (*p*_inf_, panels A and C) and the time to 100 infectious viruses (*t*_100_, panels C and D) with the initial number of viruses (*V*_0_) and the relative duration of the eclipse phase (*T_m_*, Equation (4)) as predicted by stochastic and deterministic simulations of the mathematical model (Equations (1)–(3)). In panels A and B, points represent values from stochastic simulations and dashed lines are analytical predictions (Equations (13)). Black triangles in panel B show the probability of infection for budding and bursting modes of virus production by infected cells as calculated in Pearson *et al.* [22]. In panels C and D, points are the results from the simulations and solid lines are the predictions from deterministic solutions of the model. Parameters for the simulations are given in Table 1 with values for the maturation rate *m* varied between 0.7 day^−^^1^ and 5 day^−^^1^ with *δ_I_* being adjusted to satisfy constrains as described in the text.

First, stochastic simulations predicted shorter average times to 100 viruses than the deterministic simulations, independently of the mode of virus production (Figure 3); the difference could reach 1 day for a particular set of parameters (Figure 4D). Second, the mode of virus production had little influence on the time to 100 viruses (Figure 3 and Figure 4), in contrast with a previous model [22]. Third, the mode of virus production influenced the variability in the times to 100 viruses, with a burst-like virus production resulting in more variable times to detection, consistent with previous observations [22].  The higher heterogeneity in *t*_100_ between different runs in the model with bursting virus production is expected, since some cells do not survive to make viruses. Lastly, and somewhat unexpectedly given previous results [22], a burst-like mode of virus production led to a lower probability of established infection, compared to a continuous/budding production mode (0.19 *vs.* 0.35, when starting with a single infectious virion for the parameters in Table 1, see also Figure 3 and Figure 4). This stems from the assumption that cells in the eclipse phase have a non-zero chance of dying, and thus, an increase in the duration of the eclipse phase reduces the chance that the cell will transition into a state of virus production.

**Figure 5:**
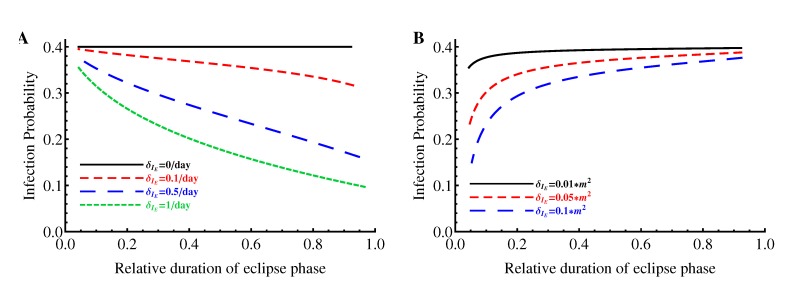
Variable dependence of the probability of infection *p*_inf_ on the relative duration of the eclipse phase, *T_m_*. Values for *c*, *βT*, and *N* are fixed according to Table 1 and the maturation rate, *m*, is varied for four different fixed values of the death rate of cells in the eclipse phase *δ_I_* (panel A) or when *m* and *δ_I_* are correlated (*δ_I_* = *θm*^2^, panel B). For a given pair of these parameters, the value of the death rate of virus-producing cells, *δ_I_*, is computed using Equation (10). The probability of established infection, *p*_inf_, and the relative duration of eclipse phase, *T_m_*, are found using Equations (4) and (13) , respectively.

We reasoned that the result that the probability of established infection decreases as the duration of the eclipse phase increases (or as the mode of virus production switches from budding to bursting) may depend on the particular set of parameters used in the simulations. Since we have an analytical expression for the probability of infection, *p*_inf_, and for the relative duration of the eclipse phase, *T_m_*, we explored whether our predictions from simulations hold true for a wider range of parameters. We observed that the results were dependent on how changes in a relative duration of the eclipse phase were achieved. When only the transition rate, *m*, or only the death rate of cells in the eclipse phase, *δ*_*I_E_*_, was changed, the probability of established infection always decreases with a longer duration of the eclipse phase as long as *δ*_*I_E_*_ > 0 (Figure 5A). However, if *m* and *δ*_*I_E_*_ were correlated, the opposite trend may be observed (*i.e.*, increasing the duration of the eclipse phase increases the probability of established infection if it also increases the death rate in the eclipse phase; Figure 5B). Thus, we conclude that whether the mode of virus production leads to either a higher or lower probability of established infection in general depends on the parameters of the model; however, if the parameters are uncorrelated, the bursting mode of virus production leads to a lower probability than the continuous/budding production mode as long as *δ*_*I_E_*_ > 0. If *δ*_*I_E_*_ = 0, then the mode of virus production has no influence on the probability that an infection becomes established. The latter result is in contrast with the conclusion found in simulations with the standard model [22].

Most of our observations regarding the time to 100 infectious viruses and the probability of infection remain true when the infection is initiated with one infected cell. For the continuous mode of virus production, predictions on the time to virus detection were nearly identical between the deterministic simulations and the stochastic realizations, with the difference between the two most pronounced with burst-like virus production (results not shown). Again, this is due to a more stochastic nature of virus production in the latter model. As expected, starting the simulations with one virus-producing cell led to a higher probability of established infection compared to starting with a single virus or when starting with one cell in the eclipse phase.

Since the initial viral dose is expected to have an influence on the virus dynamics, we ran simulations varying the initial number of viruses by two orders of magnitude under each parameter set (Table 1). As expected, a higher viral load increased the probability of established infection and decreased the time to 100 infectious viruses (Figure 4A,C). At larger initial viral doses, deterministic and stochastic simulations predicted a similar *t*_100_ (Figure 4C).

### 2.3. Comparing Model Predictions with Experimental Data

#### 2.3.1. The Model Does Not Accurately Predict the Change in the Virus Detection Time with Increasing Viral Dose

**Figure 6:**
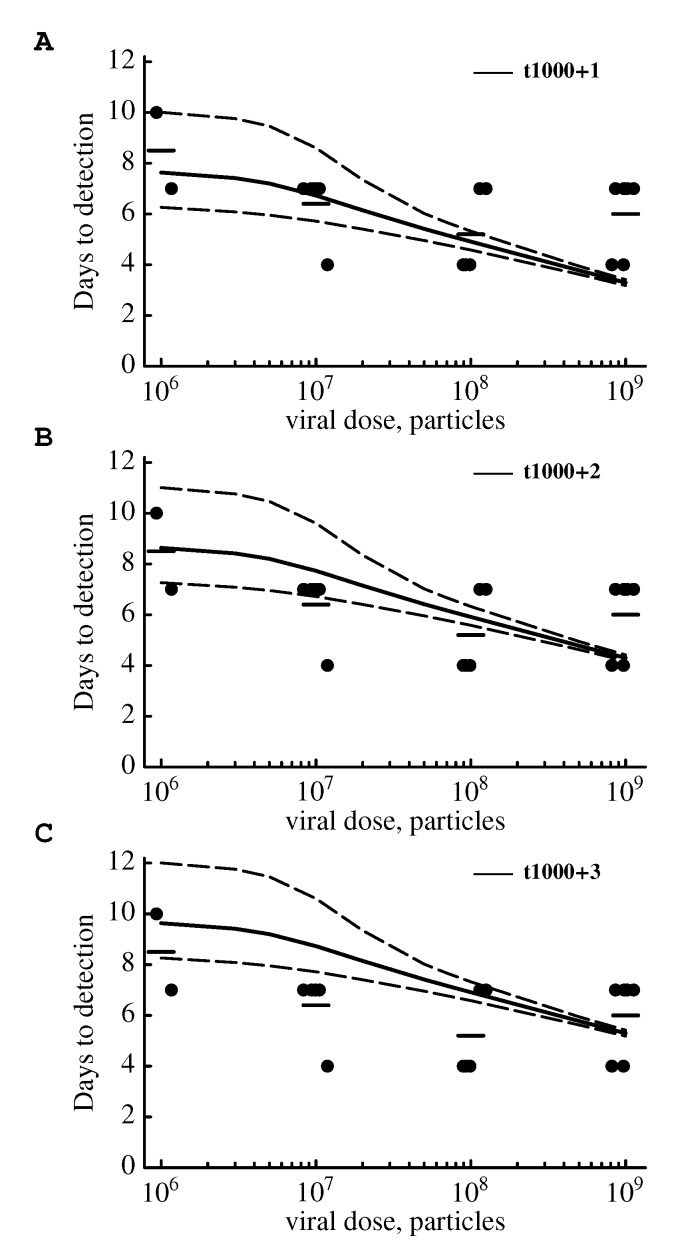
Stochastic simulations of the mathematical model do not accurately predict the change in the time to virus detection with increasing viral dose. In experiments, monkeys were challenged intrarectally with different doses of SIVmac251 and the time for virus detection in the blood was recorded [16]. We plot the time to virus detection for individual animals per dose (dots), the average of these times (bars), and the predictions from stochastic simulations of the mathematical model (solid lines) along with the 95% confidence intervals of the model predictions (dashed lines). In these simulations, we assume that exposure to 10^6^ particles leads to an infection with one infectious virus (*V*_0_ = 1), since in these experiments only a single founder virus was detected [16]. Simulations were run with parameters for an intermediate mode of virus production (Table 1). Simulations were run stochastically until 1000 infectious viruses were generated (*t*_1000_) and then deterministically for 1 (panel A), 2 (panel B), or 3 (panel C) additional days. Given the growth rate of the virus population, *r*_*o*_, (Table 1) the virus population will expand approximately 4, 18, or 76 fold in 1, 2, or 3 days, respectively.

Some qualitative information regarding the spread of SIV in the tissues of monkeys is available [5,12,38,60], but since these studies employed large initial viral doses, the applicability of those observations to low dose infections initiated by only a few virions is unclear. One recent study systematically investigated how the initial viral dose affects the time to virus detection [16]. The authors found that at the lowest initial viral dose tested (1:1000 dilution of the stock virus which is approximately 10^6^ viral particles) the time to infection was on average 8.5 days (for 2 infected animals the detection times were 7 and 10 days, Figure 6). Increasing the initial viral dose 10-, 10^2^-, or 10^3^-fold in these experiments shortened the time to virus detection to 6 days but did not change the variance in virus detection time between different animals ([16], Figure 6). Our initial simulations (see Figure 4), however, differ from these experimental results, as we predict much shorter times of virus detection, *t*_100_ (by about 3–4 days, see Figure 4C). This is likely because our defined threshold of virus detection (100 infectious viruses) may be lower than the actual number of viruses that must accumulate in the host for the infection to be detectable with standard assays.

We ran another set of stochastic simulations for an intermediate mode of virus production (see Table 1) and calculated the time when the virus population reached a size of *V * = 10^3^ (*t*_1000_) and then ran the model deterministically for 1, 2, or 3 extra days. Effectively, this approach allowed us to to determine the time to virus detection defined as *V*_det_ = 4 × 10^3^, 1.8 × 10^4^ or 7.6 × 10^4^ infectious viruses, respectively. To explain detection times at the lowest challenge dose under this model in stochastic simulations, the number of infectious viruses in the body needs to be approximately 2 × 10^4^(Figure 6B). Importantly, the model is unable to fully explain the initially large decrease in detection time with increasing viral dose and could not accurately predict the average time as observed in the data for all doses (Figure 6). Furthermore, this model predicted small variance in the time to virus detection when animals are infected with large initial doses; while in the data, the variance in the time to virus detection was approximately independent of the initial dose (results not shown). We thus conclude that the extended standard mathematical model for the SIV/HIV dynamics does not accurately predict the change in virus detection time with the initial viral dose in experimental infections of monkeys [16]. This inability of the extended standard model to accurately predict the change in time to virus detection was not due to the specific set of parameters used for simulations, since using higher values for the burst size, *N* , virus clearance rate, *c*, and the death rate of virus-producing cells, *δ_I_*, as well as varying the rate of transition, *m*, resulted in similar disagreements between model predictions and the data (see Figure S6 in Supplement). In many of these additional simulations, the initial viral dose was higher than one and was determined using Equation (18).

#### 2.3.2. The Model Does Not Accurately Predict the Change in the Probability of Established Infection with Increasing Viral Dose

**Figure 7:**
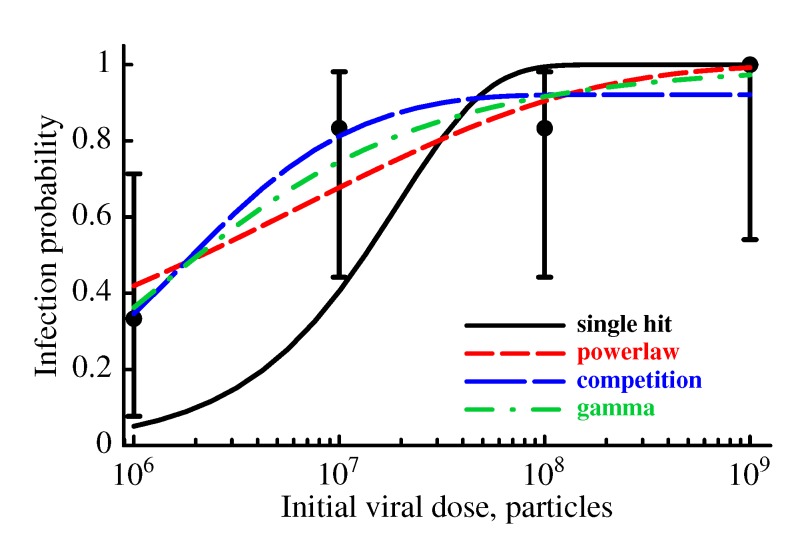
The probability of infection of monkeys with increasing SIV dose is not described well by the simple, single-hit model. We fit four different models to the experimental data of Liu *et al.* [16] using maximum likelihood. The different models are a single hit model (Equation (13)), the power law model (Equation (14)), the competition model (Equation (15)), and the gamma model (Equation (17)). The points represent the data with 95% confidence intervals calculated as Jeffreys intervals for binomial distributions [61]. Predictions of the models are given by lines. Estimated parameters of the models are: single-hit (*λ* = 5.2 × 10^−^^8^), powerlaw (*λ* = 6.7 × 10^−^^3^ and *n* = 0.32), competition (*λ* = 0.08, *α* = 5.7 × 10^−^^7^), and gamma (*λ* = 7.3 × 10^−^^7^, *σ* = 1.0 × 10^−^^6^). All models with the exception of the single-hit model describe the data with good quality as judged by the *χ*^2^ test. The powerlaw and gamma models describe the data significantly better than the single-hit model based on likelihood ratio test (*p* < 0.01). The powerlaw, competition, and gamma models describe the data with similar quality as judged by AIC [62].

Our mathematical model makes a strong prediction that the probability of an animal to become infected should increase exponentially with the initial viral dose (Figure 4 and Equation (13)). Indeed, Liu *et al.* [16] found that, as one would expect, exposure of animals to higher viral doses resulted in a higher fraction of monkeys being infected ([16], Figure 7). Interestingly, at the smallest dose tested (10^6^ viral particles), 2 out of 6 animals became infected while at 100 fold higher doses, only 4 out of 6 animals became infected (Figure 7). The standard mathematical model for SIV/HIV dynamics assumes that individual viruses do not compete within the host, and therefore, the probability of established infection of the animal should be a monotonically increasing function of the dose (Equation (13)). As discussed previously, this was the single-hit model case. When our model was fit to the experimental data using maximum likelihood, it was clear that the model is not consistent with the data (single-hit curve in Figure 7; *p* = 0.006, *χ*^2^ test). Therefore, we investigated whether three alternative models (power law model (Equation (14)), competition model (Equation (15)), and gamma model (Equation (17)) were able to explain these data.

In the first alternative model, the power law model, when infection starts with more than 1 virus, we assume that the probability of infection of the animal is not multiplicative; and instead, is nonlinearly proportional to the initial dose at small doses (Equation (14)). This dependence could potentially arise if there is competition between different viruses for access to target cells or if the number of viruses starting the infection is not linearly proportional to the initial viral dose. This model explains the experimental data significantly better than the single-hit model (Figure 7, *p* < 0.0003, likelihood ratio test).

Because the power law model is phenomenological, we also considered a competition model, in which the probability of established infection declines with the number of viruses transmitted to the host. The competition model is able to explain the data extremely well (Figure 7). Interestingly, the competition model predicts a probability of established infection less than one, even at very large viral doses. This feature may provide a basis to reject the competition model if it is found that inoculations with high doses always result in infection.

The third and final alternative model, the gamma model, assumes that the single-hit model is correct, but that there is a distribution in susceptibility of animals to viral infection (Equation (17)). As a result, the increase in the fraction of infected animals is slower than predicted by the single-hit model, because at low doses *susceptible* hosts are predominantly infected while very high viral doses are needed to infect *resistant* animals. If the distribution of susceptibilities is given by the gamma distribution, this model explains the experimental data extremely well (Figure 7), and significantly better than the single-hit

model (*p* < 0.0003, likelihood ratio test). However, the gamma model requires a highly variable susceptibility to infection between different animals since *σ* > *λ* (Figure 7) which in turn implies high variability in the parameters determining the probability of infection. Taken together, the consistency of the extended standard model with the data on infection probability of rhesus macaques with variable doses of SIV depends on additional assumptions such as high variability in the susceptibility of animals to the infection or nonlinear relationship between initial dose and the number of viruses initiating the infection.

## 3. Discussion

Mathematical models of HIV/SIV infection dynamics have been developed to address a number of important questions. Much of this work has focused on the immune response and establishment of the viral set point [30,63,64,65]. Others have focused on the early dynamics, evolution, and transmission rates of the virus [66,67,68,69,70]. Two recent studies investigated early HIV infection by stochastically simulating differential equation models with two modes of virus production by infected cells: continuous/budding and bursting [22,23]. Both cell types release the same average number of virions, *N*; however, in the first model, infected cells continuously release the virus until death, while in the second model, infected cells release the virus instantly, all at once, upon cell death. Although deterministic simulations of these two models are numerically identical, these previous studies suggest that stochastic behaviors are substantially different, with the bursting mode of production leading to a higher probability of established infection and to a longer time to infection detection than the continuous/budding mode of virus production [22,23].

In this work, we have extended these previous studies by explicitly varying the duration of the phase in which infected cells do not release virus (cellular eclipse phase). We find that for most tested parameters, the mode of virus production has a minimal effect on the time to viral detection but does influence the probability of established infection, *p*_inf_, with the burst-like mode of production resulting in a lower *p*_inf_. The latter result arises because under burst-like virus production, a cell may die before starting virus production. This aspect was different in previous models [22,23], where the death of the infected cell in the bursting model always led to virus release. In the special case when there is no death in the eclipse phase (*i.e.*, *δ*_*I_E_*_ = 0), there appears to be a stronger dependence of the time to virus detection on the mode of virus production, with the bursting mode resulting in a longer time to detection; however, in that case, the mode of virus production then had no influence on the probability of infection (see Equation (12) and Figure S5 in Supplement). Our results also demonstrate that the type of initiating infectious agent (virus particles or infected cells) and the initial load of the agent has a major influence on the probability of infection. In particular, in our simulations, starting with 10 infected cells nearly always resulted in an established infection (Figure S7 and [23]).

Our modeling approach also allows investigation of virus dynamics which do not result in productive infection; specifically, we can calculate the time to viral extinction. Across modes of virus production, if the infection is initiated by a single agent (virus or infected cell), most nonproductive infections last for less than one day (Figure S8). However, if the initial load is increased 10 fold, nonproductive infections can last up to a week, especially if the mode of virus production is bursting (Figure S9). This result may suggest that exposure to SIV or HIV, even in cases when it does not result in infection, can lead to prolonged virus dynamics and can potentially immunize exposed individuals [71,72].

An important advance of our analysis compared to previous theoretical studies is the comparison of predictions of the mathematical model with experimental data. Such comparison is not without some caveats. In particular, our model makes the assumption that early virus dynamics occurs in a well-mixed system, which means that infection should be detected experimentally as soon as the viral titers reach a particular threshold value. It is, however, difficult to know what that value should be *a priori*. We defined the virus detection threshold *V*_det_ as the virus density reached in our simulations with the lowest initial viral dose by *t*_det_ = 8.5 days since infection as was observed in experimental data ([16], Figure 6). The actual value of *V*_det_ was dependent on the model parameters, and in particular, *V*_det_ was directly proportional to the value of the viral burst size *N* (Figure S2 in Supplement). If *N* = 5 × 10^4^ [57], *V*_det_ ≈ 5 × 10^7^. It is interesting to compare this empirical estimate to a value calculated using basic anatomical properties of monkeys. Current conventional sequencing methods allow detection of a SIV/HIV infection when there is about 200 copies of viral RNA per mL of plasma, which implies *V* = 100 viral particles per mL of plasma (since each virion contains 2 RNA molecules). Assuming that the same virus concentration occurs in total body fluids and that during the first 10 days of infection there is very little cell-associated virus, the total amount of virus is *V w* where *w* is the extracellular fluid volume (EFV). We could not locate a good estimate for *w* in rhesus monkeys, but observed that monkeys weigh about 5–7 kg or 10 times less than humans. In humans, an estimate for EFV is 13.5 L [73], so we estimate *w* = 1.4 L for the monkey. Thus, the total number of viral particles in the whole body at the limit of detection in the blood is about *V_T_* = 100 viruses/mL × 1.4 × 10^3^ mL = 1.4 × 10^5^ viruses. This is lower than our theoretically calculated value (*V*_det _≈ 5 × 10^7^ viruses) suggesting that the actual burst size *N* ≈ 350 during early virus dynamics is smaller than was suggested by the *in vivo* study [57], or that only a small fraction of viruses is infectious (350/(5 × 10^4^) = 0.7%).

Another assumption of the model is that the duration of the eclipse phase is exponentially distributed. While the same assumption has been made in many previous models [30,31,32], it is a clear simplification. The actual distribution of the eclipse phase of infected cells *in vivo* is not well known but it is likely that there must be some minimal delay between the infection of a cell and virus production [54,74,75]. Following previous work [76,77,78], we formulated a mathematical model which assumes a fixed delay between infection of a cell and virus production (see Equations (S19) and (S20) in Supplement). Deterministic simulations of this model expectedly predict exponential increase in the virus population size after the initial transient (Figure S10 in Supplement). Furthermore, model solutions predict a monotonic decrease in the time to virus detection with increasing initial viral dose which is very similar to the predictions of the model with exponentially distributed eclipse phase (compare Figures S6 and S11 in Supplement). As in the model with exponentially distributed eclipse phase, the model with a fixed duration eclipse phase is unable to accurately explain the the relatively constant level of the time to virus detection at high (>10^6^) initial viral doses (Figure S11 in Supplement).

One of the major take-home messages of our analysis is that the mode of virus production by infected cells plays a minor role in determining early SIV/HIV dynamics, so in many cases it can be ignored by allowing virus-infected cells to continuously produce the virus. Then if *m* → *∞* and *N* ≫ 1, for infections initiated by a single virus, the virus dynamics simplify to either virus extinction with the probability *c*/(*βT* + *c*) or exponential growth of the virus population at the replication rate *r* + *δ_I_* and death rate *δ_I _*. These dynamics can be then modeled as a simple linear random birth-death process [79,80]. Our analysis demonstrates that the extended standard mathematical model is too simple to accurately explain the change in the time to virus detection with increasing viral doses (Figure 6). It is possible that the discrepancy between the model and the data is due to relatively poor sampling of the viral load during early infection or because the initial number of viruses, starting the infection, is not proportional to the initial viral dose. Alternatively, this discrepancy could be due to some important biological components that are missing from the model.  In particular, the model assumes unlimited supply of target cells for virus replication, while several studies have highlighted the spatial heterogeneity of uninfected and infected cells, and the loss of many CD4 T cells during early infection [5,81,82,83,84]. Furthermore, it has been suggested that systemic virus infection occurs not from the gut tissues but from lymph nodes [85], and viral spread from the gut to lymph nodes was not included in our model.  Finally, we assumed that parameters for virus dynamics are identical between different animals, but the observed variability in the time to virus detection even at high initial viral doses could be a function of differences in susceptibility between individual animals. Indeed, a model in which the probability of virus extinction is varied between animals allowed for a good description of the data on the probability of established infection with increasing viral doses (Figure 7). Alternatively, there may be competition between viruses replicating at different sites of the gut.  Such competition may impact the change in the probability of infection with increasing viral doses (Figure 7) and could potentially influence the change in the time to virus detection with increasing viral doses (Figure 6).  Future studies should investigate these alternative hypotheses.

Variability in parameters for virus dynamics between individual animals could be also the source of variability in the observed times to virus detection. Analysis of experimental data on *in vitro* HIV dynamics revealed large variability in the times when individual infected cells start producing the virus [74,75,86]. Therefore, to investigate how variability in model parameters may impact the virus dynamics and the time to virus detection we simulated virus dynamics in the basic model (Equations (1)–(3)) using parameters for the intermediate mode of virus production. For every run we used a value for the virus growth rate *r* sampled from the distribution of SIV population growth rates observed in Liu *et al.* [16] (log-normal distribution with average $\bar{\log_{10}r}$ = 0.16 and standard deviation *σ*_log_10_*r*_ = 0.07). The analysis shows that variability in the virus replication rates translates into variability in the time to virus detection with larger variability in times to detection at smaller initial doses (Figure S12 in Supplement). However, this experimentally observed variability in the virus replication rates was still unable to accurately predict a relatively constant time to virus detection at high initial viral doses. We expect that combining stochastic simulations with variability in model parameters will lead to even greater variance in the time to virus detection at low initial viral doses which is inconsistent with experimental data of Liu *et al.* [16].

## 4. Materials and Methods

### 4.1. Implementing Stochastic Simulations

To simulate our system stochastically, we used the Gillespie algorithm [59], implemented with the GillespieSSA package in R (www.r-project.org). In the Gillespie algorithm, the system of differential equations is organized into a state-change matrix that describes each of the possible occurrences in the system and the rate at which they occur: a virus can infect a target cell, thereby becoming an infected cell in the eclipse phase, such a cell can become a productively infected cell, a productively infected cell can release viruses, or any of the species can die. For every time step, two random numbers are generated and used to determine (1) which event will occur next and (2) the amount of time that will pass until it occurs.

Pearson *et al.* [22] used 32 infected cells as a threshold for an established infection. We chose instead to track virus numbers, a quantity that is generally measured in experiments. The number of infectious viruses at which the infection becomes detectable was determined by matching model predictions to experimental data for different parameter sets. In some simulations, we used *V*_det_ = 100 or 1000 infectious viruses because at this value the dynamics of the populations in the model became relatively deterministic. At *V* *∼* 10^3^, the dynamics of the populations in our model are nearly deterministic since the coefficient of variation, for example, in virus number is only $1/\sqrt{10^{3}}=0.032$ or 3%, which is fairly small. Dynamics of cell populations at later times were simulated deterministically using the ode routine in library deSolve in R.

### 4.2. Statistical Tests

To compare the nested models we use the likelihood ratio test [62,87]. In cases when the compared models are not nested, we use AIC [62]. To determine the quality of the model fits to experimental data, we use *χ*^2^ test.

## 5. Conclusions

The first week of infection is a crucial time for viral growth and dissemination but is extremely difficult to study *in vivo*. We believe that our study contributes to a better understanding of the process of transmission and early infection with SIV (and possibly HIV). Our analysis suggests that simple, one-compartment models are not consistent with the data on SIV dynamics during early stages of infection. More detailed models will be needed to provide a better description of early virus dynamics, and testing and rejecting different versions of such models will require very detailed quantitative experimental data.

## Supplementary Files

Supplementary File 1
