# Supplementary material for: Simple Mathematical Models Do Not Accurately Predict Early SIV Dynamics
Source: Viruses. 2015 Mar 13;7(3):1189–217. doi: 10.3390/v7031189 (PMC4379566; doi:10.3390/v7031189)
Supplement: Supplementary File 1 [file viruses-07-01189-s001.pdf]

Supplement

Fitting infection data using likelihood

To fit theoretical formulas on the change in probability of infection with viral dose we use maximum likelihood approach [87]. For a particular trial of infection of a host with an initial dose of the virus V_0, the likelihood that i animals are infected out of a total of N animals is given by the binomial distribution

$$L(i, N|\lambda, n, V_0) = \left(\begin{array}{c} N \\ i \end{array} \right) [p_{\text{inf}}(V_0)]^i [1 - p_{\text{inf}}(V_0)]^{N-i}, \tag{S1}$$

where p_{inf} is the probability of infection (e.g., eqn. (13)). When an infection trial was done for multiple initial viral doses, likelihoods of infection were multiplied and best fit parameters were found by maximizing the likelihood, L, or log-likelihood, $\mathcal{L}$:

$$\mathcal{L} = \log L = \sum_{V_0} i \log p_{\text{inf}}(V_0) + \sum_{V_0} (N - i) \log(1 - p_{\text{inf}}(V_0)) + \text{const.} \tag{S2}$$

Calculating the dynamics of infected cells

To determine the impact of variability in the burst size N on the total virus found at a specific time t (e.g., time of virus detection t_{det}), it is useful to rescale the extended model for virus dynamics by defining $v = V/N$. Then the model becomes:

$$\frac{\mathrm{d}v}{\mathrm{d}t} = \delta_I I - (\beta T + c)v, \tag{S3}$$

$$\frac{\mathrm{d}I_E}{\mathrm{d}t} = \beta T N v - (m + \delta_{I_E}) I_E, \tag{S4}$$

$$\frac{\mathrm{d}I}{\mathrm{d}t} = m I_E - \delta_I I. \tag{S5}$$

Under the condition that $c \gg \beta T$, the product $\beta T N$ is proportional to the observed growth rate of the virus population r (see eqn. (9) in Main text). Then the dynamics of the rescaled model (eqns. (S3)–(S5)) is independent of the burst size N. Therefore, this analysis illustrates that under biologically reasonable conditions the time to virus detection is determined strictly by the dynamics of infected cells, and the amount of free virus at the time of virus detection scales linearly with N.

Given that asymptotically the dynamics of the model follow exponential growth or decay, we sought to determine the value for the initial number of productively infected cells I^* that we expect if population size were to grow exponentially from time 0 (Figure S1). Because the original model is rather complicated, we performed our analysis with the standard model found by letting $m \to \infty$ in eqns. (S3)–(S5).

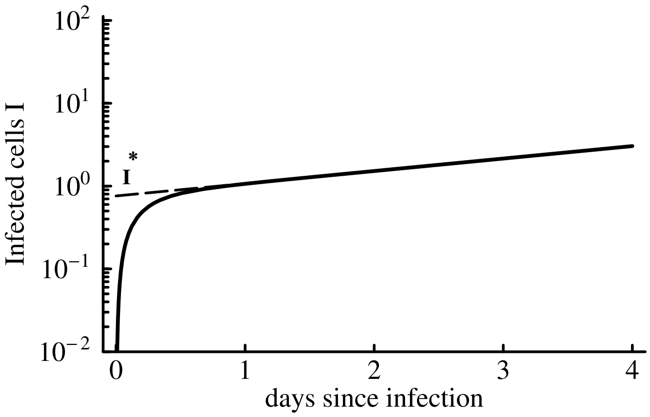

Figure S1: Schematic representation of the dynamics of productively infected cells and the calculation of the initial number of infected cells, I^*, if the number of infected cells was increasing exponentially at a rate r.

In the standard model, the rate of growth of the virus population is simply $r = \delta_I \left(\frac{\beta T N}{c+\beta T} - 1\right)$, and after re-arranging variables and assuming that $N \gg (1+\frac{r}{\delta_I})$ we find that $\beta T = \frac{c}{N}\left(1+\frac{r}{\delta_I}\right)$. Replacing βT in eqn. (S3), we find the standard rescaled model as

$$\frac{\mathrm{d}I}{\mathrm{d}t} = c\left(1+\frac{r}{\delta_I}\right) - \delta_I I, \tag{S6}$$

$$\frac{\mathrm{d}v}{\mathrm{d}t} = \delta_I I - cv. \tag{S7}$$

Asymptotic behavior of the model is determined by the eigenvalues and the predicted initial conditions (I^*, V^*) are given by eigen vectors. Eigenvalues of the model are found in equation

$$\begin{vmatrix} -\delta_I - \lambda & c(1+r/\delta_I) \\ \delta_I & -c-\lambda \end{vmatrix} = 0$$

and eigenvalues are

$$\lambda_{1,2} = \frac{\pm\sqrt{(c+\delta_I)^2 + 4rc} - (c+\delta_I)}{2} \tag{S8}$$

Asymptotically when $c \gg \delta_I$, $\lambda_1 \approx r$ and $\lambda_2 \approx -c$ as expected. Recalling that $V_0 = \frac{a_0 N \delta_I}{r}$ (see eqn. (18)), the asymptotic behavior of the dynamics of infected cells is

$$I(t) \approx I^* \mathrm{e}^{\lambda_1 t} = \frac{a_0(c+\lambda_1)(c+\lambda_2)}{r(\lambda_2-\lambda_1)}\mathrm{e}^{\lambda_1 t} \approx a_0\left(1+\frac{\delta_I}{r}\right)\mathrm{e}^{rt}, \tag{S9}$$

This result further demonstrates that the dynamics of the infected cells are only determined by the growth rate of the virus population, death rate of productively infected cells, and the probability of infection of the animal. At the limit of the rapid removal of free viruses ($c \to \infty$), the dynamics of scaled virus particles is also independent of N, $v(t) \approx \frac{\delta_I I^*}{c} \mathrm{e}^{rt}$. Then the dynamics of absolute number of virus particles is simply $V(t) \approx \frac{a_0 N \delta_I (r+\delta_I)}{cr} \mathrm{e}^{rt}$, and therefore, the virus density at which infection becomes detected is directly proportional to the burst size N. Direct scaling of the virus number at which infection becomes detected with N is illustrated in Figure S2.

Calculating the probability of extinction

For the model eqns. (1)–(3) with three species V, I_E and I, we denote $P_{V_0,I_{E0},I_0;V^*,I_E^*,I^*}(t)$ as the transition probability that $(V(t), I_E(t), I(t)) = (V^*, I_E^*, I^*)$ at time t with the initial condition $(V(0), I_E(0), I(0)) = (V_0, I_{E0}, I_0)$. Given the initial distribution on three species, $p_{V_0,I_{E0},I_0}(0)$, the probability distribution at time t can be expressed as

$$p_{V^*,I_E^*,I^*}(t) = \sum_{V_0,I_{E0},I_0=0}^{\infty} P_{V_0,I_{E0},I_0;V^*,I_E^*,I^*}(t) p_{V_0,I_{E0},I_0}(0). \tag{S10}$$

Since all species are of finite sizes, $P_{V_0,I_{E0},I_0;V^*,I_E^*,I^*}(t) \to 0$ and $p_{V_0,I_{E0},I_0}(0) \to 0$ as $V_0, I_{E0}, I_0 \to \infty$.

Following a similar procedure as shown in [88], we can derive the backwards Chapman-Kolmogorov differential equation for $P_{V_0,I_{E0},I_0;V^*,I_E^*,I^*}(t)$ [89]:

$$\begin{aligned}
&\frac{dP_{V_0,I_{E0},I_0;V^*,I_E^*,I^*}(t)}{dt} \\
&= mI_{E0}(P_{V_0,I_{E0}-1,I_0+1;V^*,I_E^*,I^*} - P_{V_0,I_{E0},I_0;V^*,I_E^*,I^*}) + \delta_{I_E} I_{E0}(P_{V_0,I_{E0}-1,I_0;V^*,I_E^*,I^*} - P_{V_0,I_{E0},I_0;V^*,I_E^*,I^*}) \\
&+ \delta_I I_0(P_{V_0,I_{E0},I_0-1;V^*,I_E^*,I^*} - P_{V_0,I_{E0},I_0;V^*,I_E^*,I^*}) + \delta_I N I_0(P_{V_0+1,I_{E0},I_0;V^*,I_E^*,I^*} - P_{V_0,I_{E0},I_0;V^*,I_E^*,I^*}) \\
&+ cV_0(P_{V_0-1,I_{E0},I_0;V^*,I_E^*,I^*} - P_{V_0,I_{E0},I_0;V^*,I_E^*,I^*}) + \beta T V_0(P_{V_0-1,I_{E0}+1,I_0;V^*,I_E^*,I^*} - P_{V_0,I_{E0},I_0;V^*,I_E^*,I^*})
\end{aligned} \tag{S11}$$

with initial condition $P_{V_0,I_{E0},I_0;V^*,I_E^*,I^*}(0) = \theta_{V_0 V^*} \theta_{I_{E0} I_E^*} \theta_{I_0 I^*}$ (here θ_{ij} is the Kronecher delta function). The probability generating function $G_{V_0,I_{E0},I_0}(x, y, z; t)$, given initial condition $(V(0), I_E(0), I(0)) = (V_0, I_{E0}, I_0)$, is defined as

$$\begin{aligned}
G_{V_0,I_{E0},I_0}(x, y, z; t) &= E[x^{V(t)} y^{I_E(t)} z^{I(t)} \big| V(0) = V_0, I_E(0) = I_{E0}, I(0) = I_0] \\
&= \sum_{V^*,I_E^*,I^*=0}^{\infty} P_{V_0,I_{E0},I_0;V^*,I_E^*,I^*}(t) x^{V^*} y^{I_E^*} z^{I^*}.
\end{aligned}$$

By exploiting the branching property

$$G_{V_0,I_{E0},I_0}(x, y, z; t) = (G_{1,0,0}(x, y, z; t))^{V_0} (G_{0,1,0}(x, y, z; t))^{I_{E0}} (G_{0,0,1}(x, y, z; t))^{I_0},$$

we can reduce the infinite-dimensional differential equations for $G_{V_0,I_{E0},I_0}(x,y,z;t)$ to three equations. For convenience, we denote $G_1(x,y,z;t) = G_{1,0,0}(x,y,z;t)$, $G_2(x,y,z;t) = G_{0,1,0}(x,y,z;t)$ and $G_3(x,y,z;t) = G_{0,0,1}(x,y,z;t)$.

$$\begin{aligned}
\frac{dG_1(x,y,z;t)}{dt} &= c(1 - G_1(x,y,z;t)) - \beta T(G_1(x,y,z;t) - G_2(x,y,z;t)),\\
\frac{dG_2(x,y,z;t)}{dt} &= -m(G_2(x,y,z;t) - G_3(x,y,z;t)) - \delta_{I_E}(G_2(x,y,z;t) - 1),\\
\frac{dG_3(x,y,z;t)}{dt} &= -\delta_I(G_3(x,y,z;t) - 1) - \delta_I N G_3(x,y,z;t)(1 - G_1(x,y,z;t)).
\end{aligned} \tag{S12}$$

with initial conditions $G_1(x,y,z;0) = x$, $G_2(x,y,z;0) = y$ and $G_3(x,y,z;0) = z$.

Define $q = q_V$ as the probability that a single infectious virion does *not* initiate an HIV infection. From the definition, q_V can be obtained from $P_{V_0,I_{E0},I_0;V^*,I_E^*,I^*}(t)$ in the following way:

$$q_V = \lim_{t\to\infty} P_{1,0,0;0,0,0}(t).$$

Since $P_{1,0,0;0,0,0}(t) = G_1(0,0,0;t)$, q_V can be expressed as $q_V = \lim_{t\to\infty} G_1(0,0,0;t)$. The probability of extinction, q_V, is the $G_1(x,y,z;t)$ coordinate of the stable fixed point of the system (S12). After straightforward calculation, we get the expression of q_V:

$$q_V = \begin{cases} 1, & \beta T \le \frac{c(m+\delta_{I_E})}{m(N-1)-\delta_{I_E}},\\ \frac{(\delta_{I_E}+m)(\beta T + c + cN) + N\beta T\delta_{I_E}}{N(c+\beta T)(\delta_{I_E}+m)}, & \beta T > \frac{c(m+\delta_{I_E})}{m(N-1)-\delta_{I_E}}. \end{cases} \tag{S13}$$

where $m(N-1) - \delta_{I_E} > 0$. So, the probability of infection, given that a single replicating virion initiates it, is given as $1 - q_V$. Note that the condition $\frac{\beta T(m(N-1)-\delta_{I_E})}{c(m+\delta_{I_E})}$ comes from the stable condition of the fixed point of system (S12). From this condition, we can define the basic reproductive number R_0 as

$$R_0 = \frac{\beta T(m(N-1) - \delta_{I_E})}{c(m + \delta_{I_E})} = \frac{\beta T}{c}\left(\frac{m}{m+\delta_{I_E}}N - 1\right).$$

R_0 has clear biological meaning, and it gives the number of newly produced virions generated by one virion through infecting target cells during its lifetime.

Similarly, we define q_{I_E} (or q_I) as the probability that a single cell in the eclipse (or producing/releasing) phase does *not* initiate an HIV infection. This gives

$$q_{I_E} = \begin{cases} 1, & \beta T \le \frac{c(m+\delta_{I_E})}{m(N-1)-\delta_{I_E}},\\ \frac{N\beta T\delta_{I_E} + (\delta_{I_E}+m)(\beta T + c)}{N\beta T(\delta_{I_E}+m)}, & \beta T > \frac{c(m+\delta_{I_E})}{m(N-1)-\delta_{I_E}}, \end{cases} \tag{S14}$$

$$q_I = \begin{cases} 1, & \beta T \le \frac{c(m+\delta_{I_E})}{m(N-1)-\delta_{I_E}},\\ \frac{(\delta_{I_E}+m)(\beta T + c)}{N\beta T m}, & \beta T > \frac{c(m+\delta_{I_E})}{m(N-1)-\delta_{I_E}}. \end{cases} \tag{S15}$$

In fact, $q_{I_E} = \frac{\delta_{I_E}}{m+\delta_{I_E}} + \frac{m}{m+\delta_{I_E}} q_I$, where $\frac{\delta_{I_E}}{m+\delta_{I_E}}$ and $\frac{m}{m+\delta_{I_E}}$ are the fractions of target cells that died in the eclipse phase and that moved to the productively infected phase, respectively.

Deriving the model with a fixed delay eclipse phase

We formulate the model with a fixed delay as a combination of the ODE and PDE

$$
\begin{aligned}
\frac{\partial i_E(t,\tau)}{\partial t} + \frac{\partial i_E(t,\tau)}{\partial \tau} &= -\delta_{I_E} i_E(t,\tau), && \text{(S16)}\\
\frac{\mathrm{d}I(t)}{\mathrm{d}t} &= i_E(t,\Delta) - \delta_I I(t), && \text{(S17)}\\
\frac{\mathrm{d}V(t)}{\mathrm{d}t} &= N\delta_I I(t) - (c+\beta T)V(t), && \text{(S18)}
\end{aligned}
$$

with the boundary condition $i_E(t,0) = \beta T V(t)$. In the model Δ is the duration of the eclipse phase and other parameters are as defined in the main text. By solving eqn. (S16) by method of characteristics we find $i_E(t,\tau) = \beta T V(t-\tau)\mathrm{e}^{-\delta_{I_E}\tau}$. The total number of cells in the eclipse phase is defined as $I_E(t) = \int_0^\Delta i_E(t,\tau)\mathrm{d}\tau$. Integrating eqn. (S16) we find an equation for $I_E(t)$ and $I(t)$

$$
\begin{aligned}
\frac{\mathrm{d}I_E(t)}{\mathrm{d}t} &= \beta T V(t) - \beta T V(t-\Delta)\mathrm{e}^{-\delta_{I_E}\Delta} - \delta_{I_E} I_E(t), && \text{(S19)}\\
\frac{\mathrm{d}I(t)}{\mathrm{d}t} &= \beta T V(t-\Delta)\mathrm{e}^{-\delta_{I_E}\Delta} - \delta_I I(t), && \text{(S20)}
\end{aligned}
$$

and the dynamics of the cell-free virus is given in eqn. (S18).

Additional results of simulations

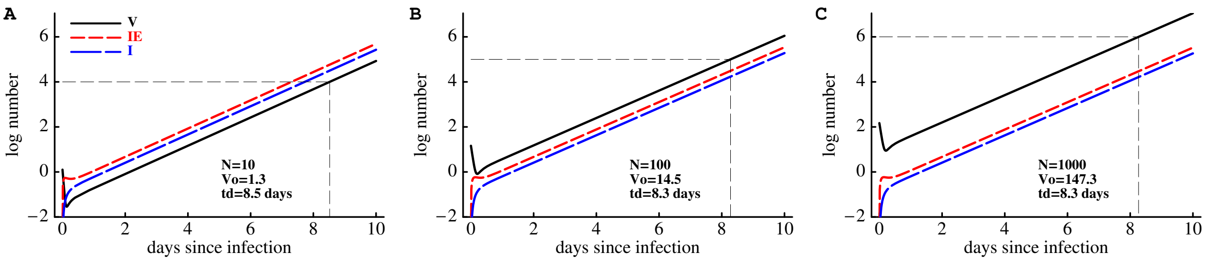

Figure S2: The burst size N influences the virus detection limit V_{det}. We run deterministic simulations of the extended mathematical model (eqns. (1)–(3)) for different values of the burst size N (values are indicated on the panels). Parameters of the model (m, d_I, d_{I_E}, and c) were fixed to values for the intermediate mode of virus production by infected cells (Table 1 in the main text), and other parameters varied to satisfy constrains on r and p_{inf}. The proposed detection limits are $V_{\text{det}} = 10^4$, 10^5, and 10^6 infectious viruses for $N = 10$, 100, and 1000, respectively.

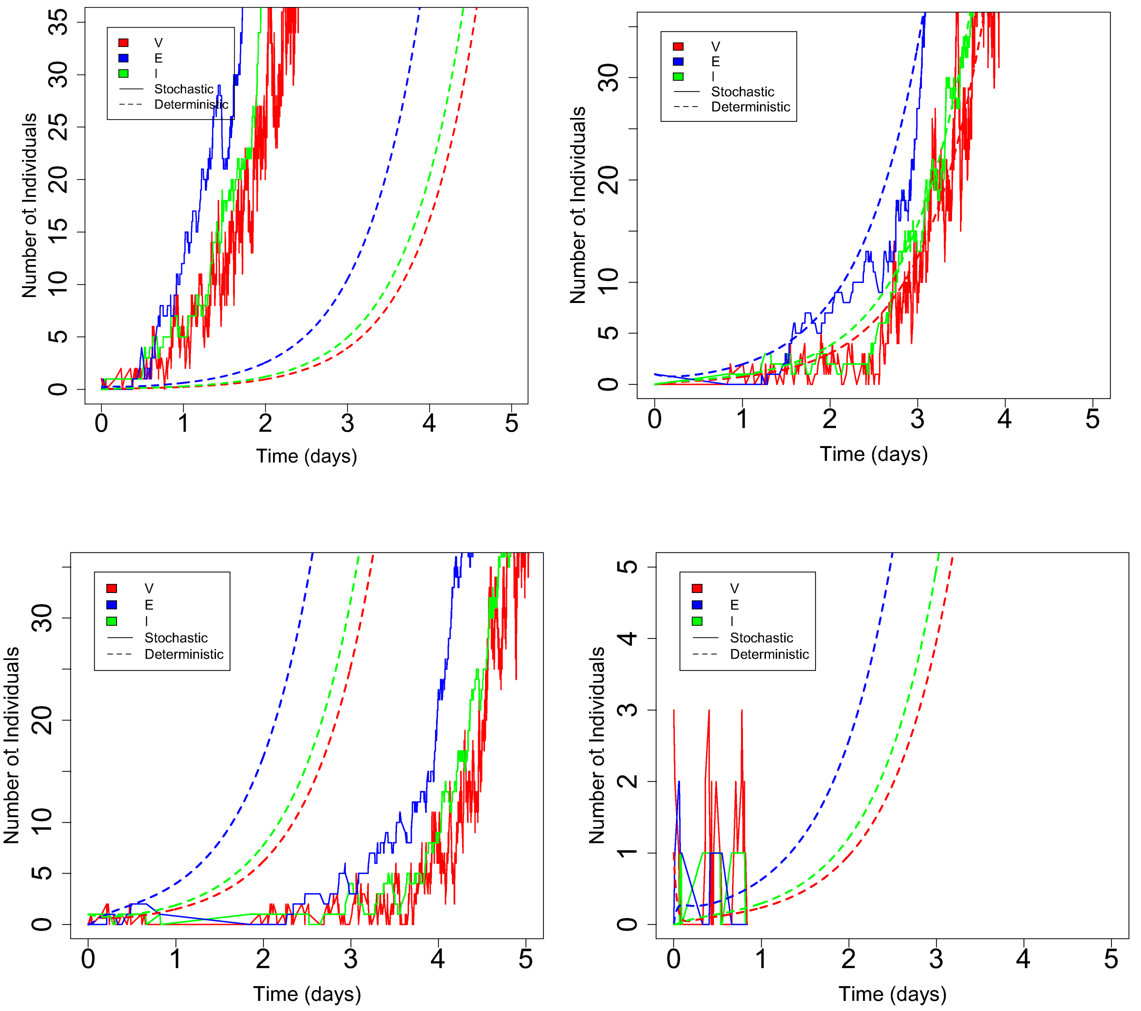

Figure S3: Several individual simulations of virus and cell dynamics as compared with the predictions of the deterministic model. Parameters for simulation are shown in Table 1 for the intermediate mode of virus production by infected cells. In these example simulations we show that stochastic virus dynamics may occur faster than that predicted by the deterministic model (top left panel), dynamics may be similar in stochastic and deterministic simulations (top right panel), or dynamics can be slower in stochastic simulations than that in deterministic simulations (bottom left panel). Also, virus can go extinct in stochastic simulations (bottom right panel).

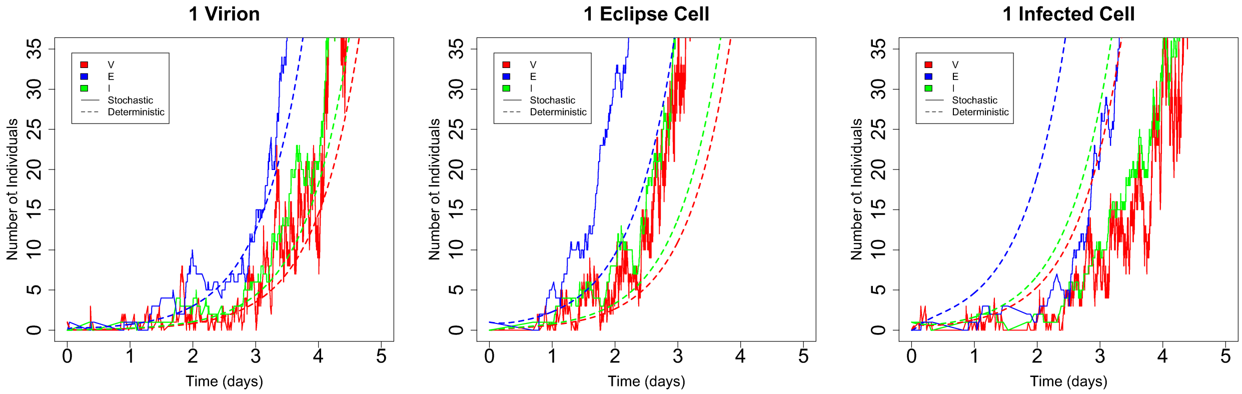

Figure S4: Dynamics of cell populations as simulated deterministically and stochastically (using Gillespie algorithm). An intermediate mode of virus production (see Table 1 in the main text) was chosen and simulations were started with one virus (left), one cell in the eclipse phase (middle), or one productively infected cell (right). The relationship between the time it takes the infection to take off exponentially illustrates the overall trend between the stochastic and the deterministic models.

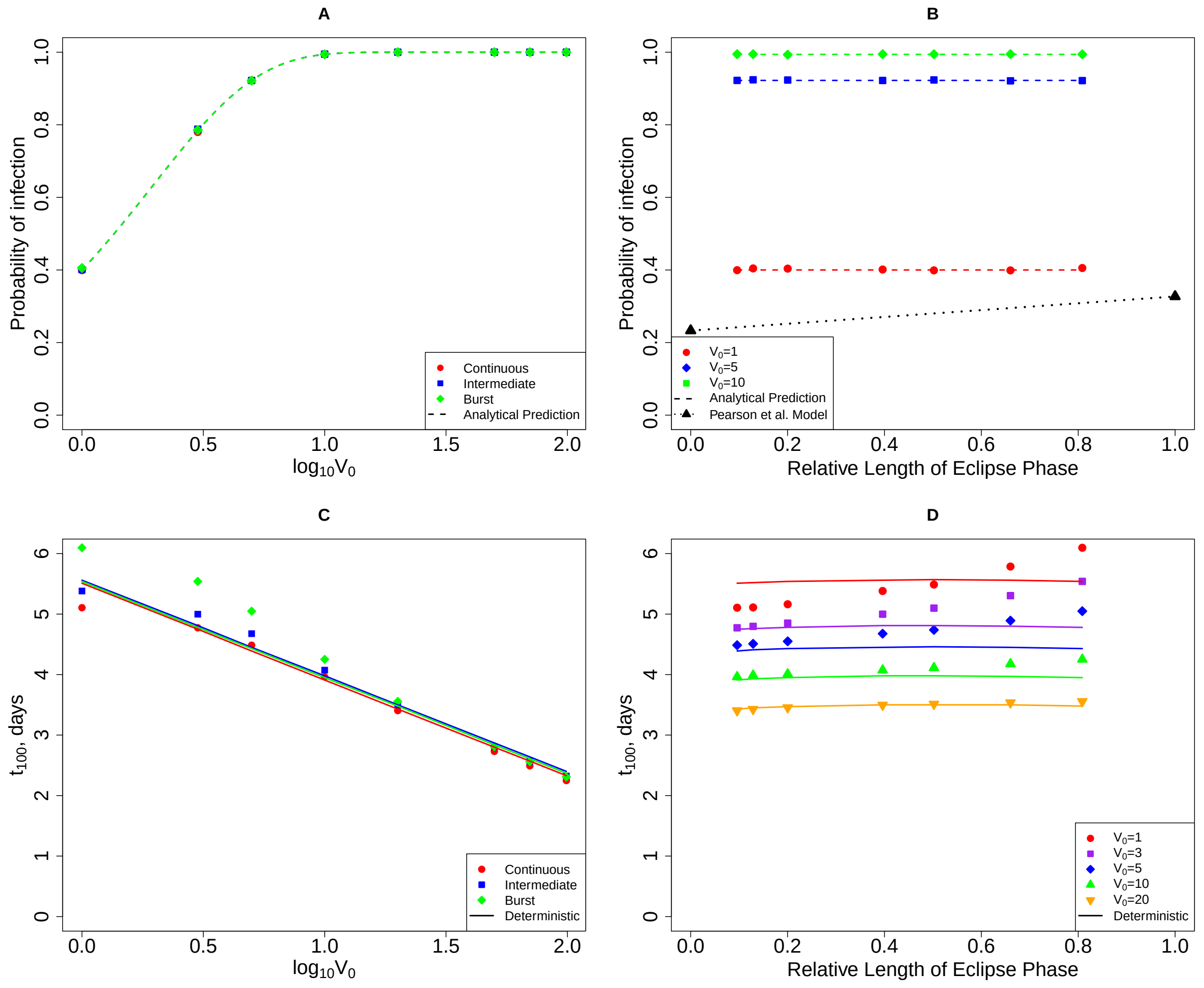

Figure S5: The probability of infection ($p_{\rm inf}$, panels A and C) and the time to virus detection (t_{100}, panels C and D) versus initial viral dose, V_0, (left column) and the relative duration of the eclipse phase, T_m, eqn. (4) (right column). In these simulations, we set $\delta_{I_E} = 0$ (compare to Figure 4 in the Main text). In panels A and B, points represent values from stochastic simulations and dashed lines are analytical predictions (eqn. (13)). In panels C and D, points represent values from stochastic simulations and solid lines are predictions from the deterministic solutions of the model.

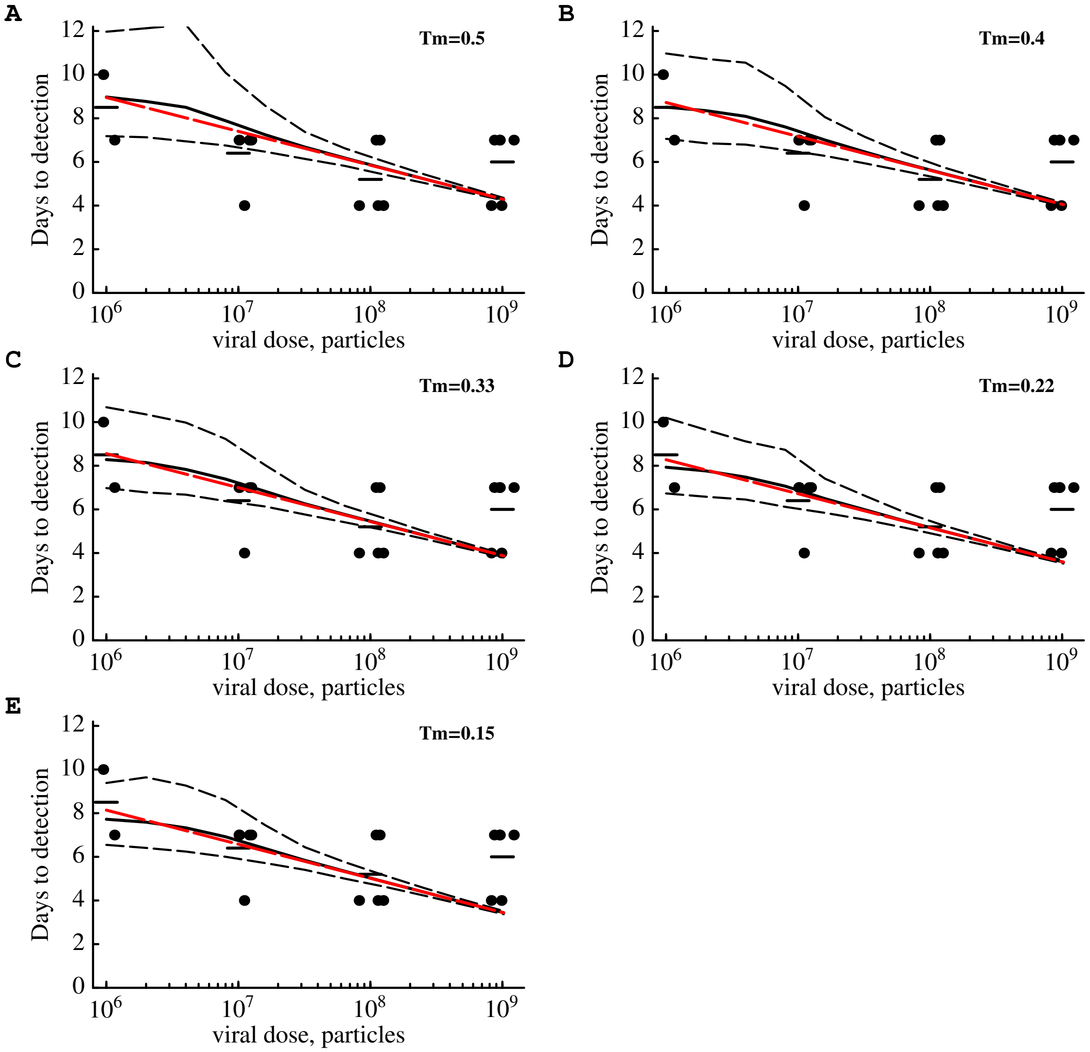

Figure S6: The model is unable to accurately predict the change in the time of virus detection with larger viral doses at different parameter values. We let $N = 100$ infectious viruses, $c = 100$ day^{-1}, $\delta_I = 1$ day^{-1}, $\delta_{I_E} = 0.5$ day^{-1}, and we vary m from 0.5 to 5 day^{-1}. Parameters βT and V_0 were calculated to satisfy constrains imposed by the data with $V_{\text{det}} = 5 \times 10^4$ infectious viruses. We ran 1000 Gillespie simulations for every parameter set. The think black lines denote the average of the stochastic simulations, and thin dashed lines are 95% confidence intervals from stochastic simulations. The thick red line is the predictions of the deterministic simulations of the model. Dots denote the time to virus detection for individual animals from Liu *et al.* [16] and bars are the average time per given dose. Interestingly, at high rate of virus clearance c, the average prediction of the stochastic simulations and deterministic simulations are nearly identical.

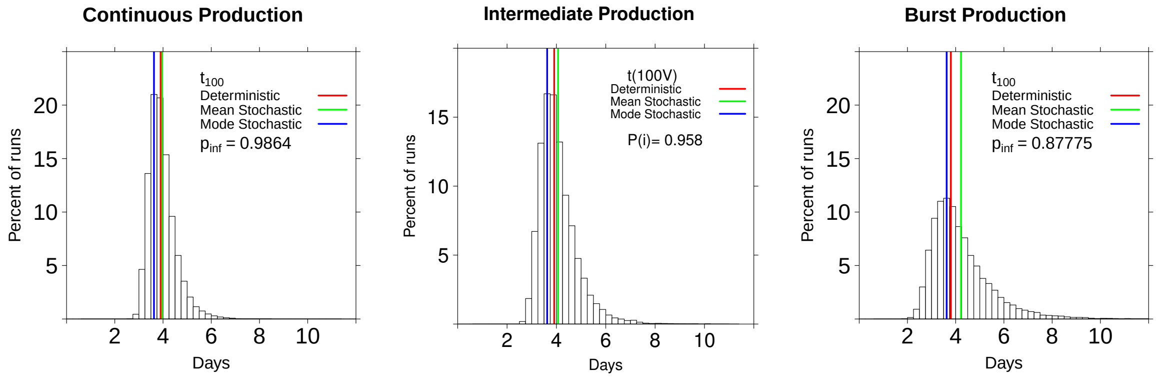

(a) Times to virus detection starting with 10 virions.

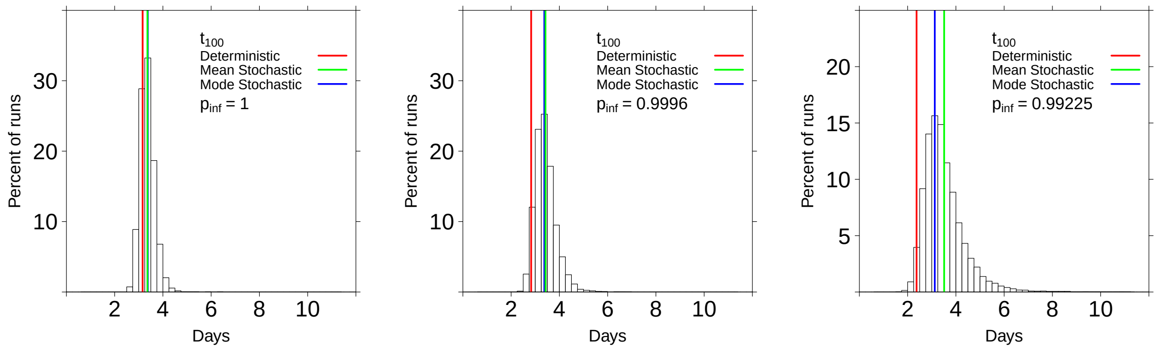

(b) Times to virus detection starting with 10 eclipsed cells.

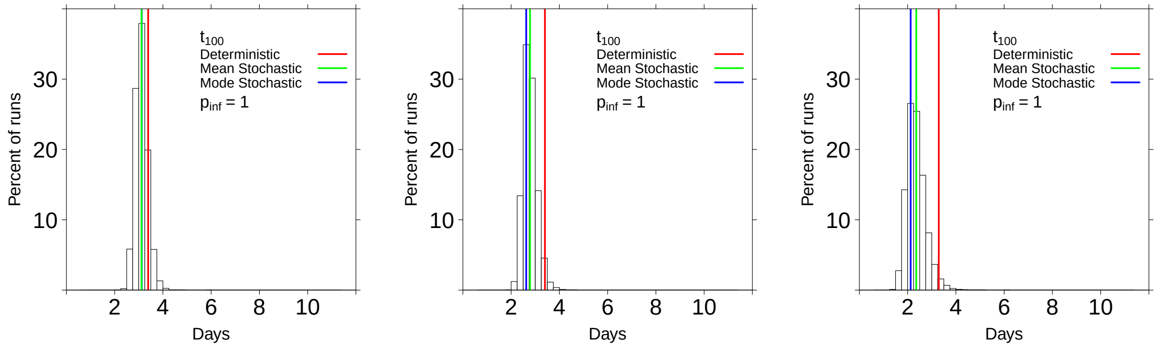

(c) Times to virus detection starting with 10 infected cells.

Figure S7: Distribution of times to 100 infectious viruses (t_{100}) with an initial dose of (a) 10 viruses, (b) 10 cells in the eclipse phase, or (c) 10 virus-producing cells. The mode of virus production used continuous/budding for the left column, intermediate for the middle column, and burst-like for the right column. We ran 20,000 simulations of the basic mathematical model using the Gillespie algorithm.

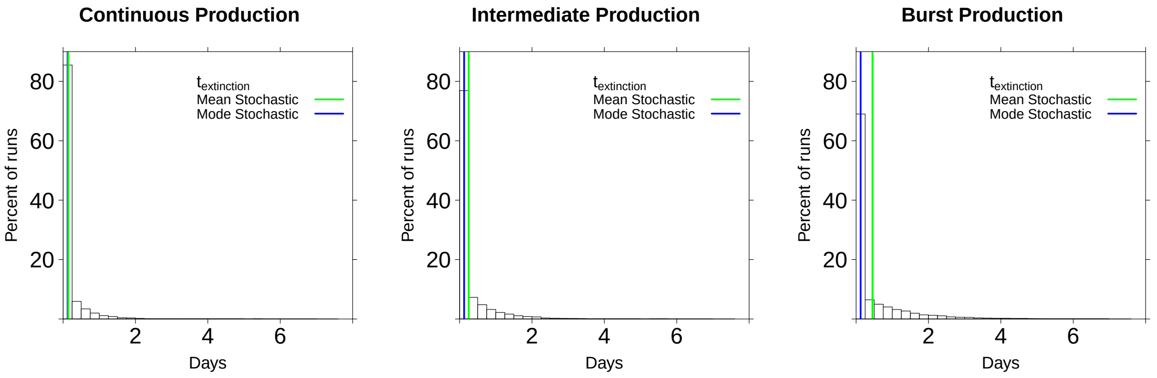

(a) Times to extinction starting with one infectious virion.

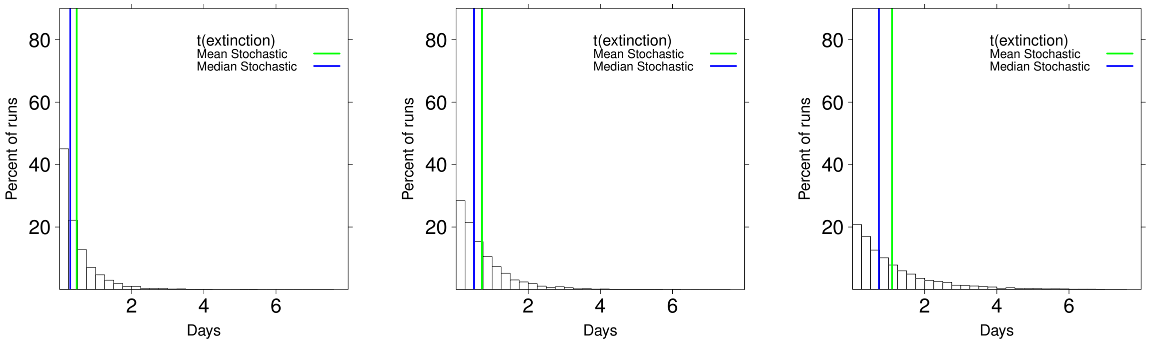

(b) Times to extinction starting with one cell in the eclipse phase.

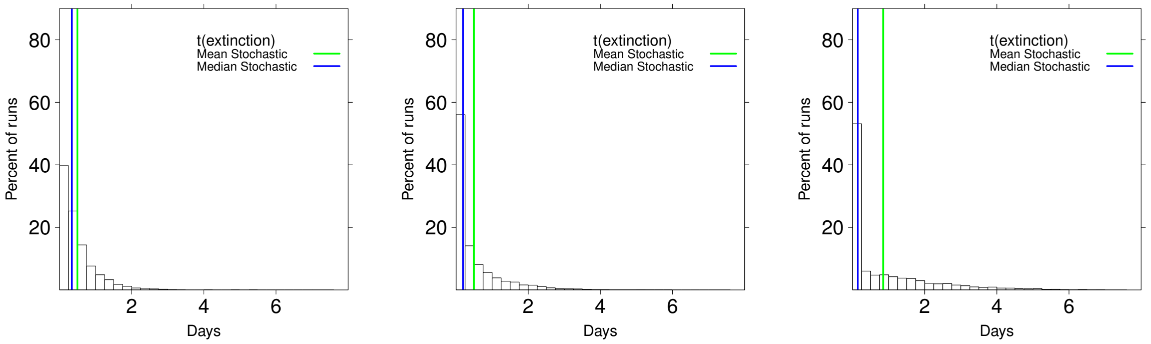

(c) Times to extinction starting with one virus-producing cell.

Figure S8: Distribution of times to virus extinction, starting with an initial load of (a) 1 virus, (b) 1 cell in the eclipse phase, or (c) 1 virus-producing cell. The mode of virus production used is continuous/budding for the left column, intermediate for the middle column, and burst-like for the right column.

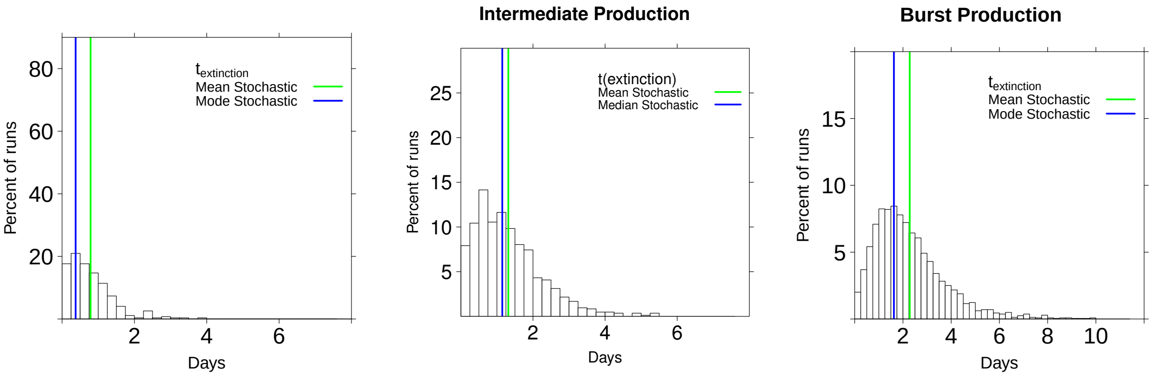

(a) Times to extinction starting with 10 virions.

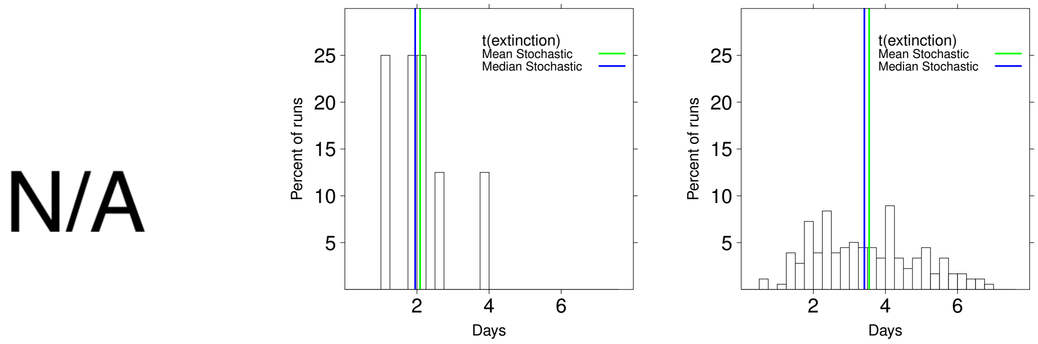

(b) Times to extinction starting with 10 cells in the eclipse phase.

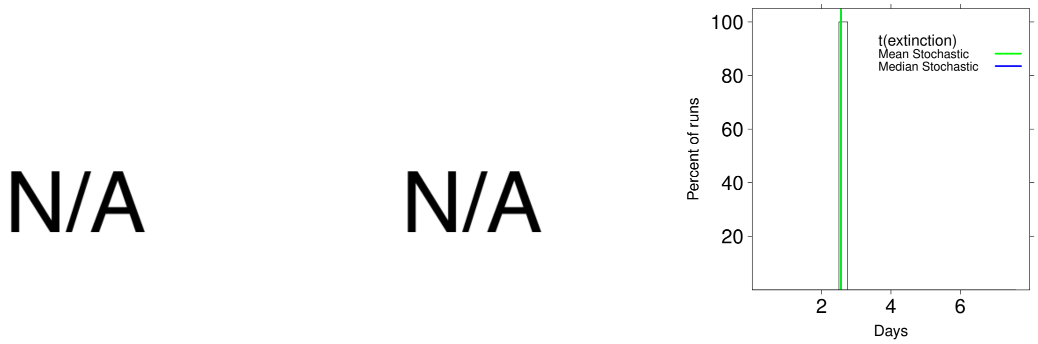

(c) Times to extinction starting with 10 virus-producing cells.

Figure S9: Distribution of times to virus extinction, starting with an initial load of (a) 10 viruses, (b) 10 cells in the eclipse phase, or (c) 10 virus-producing cells. The mode of virus production used is continuous/budding for the left column, intermediate for the middle column, and burst-like for the right column.

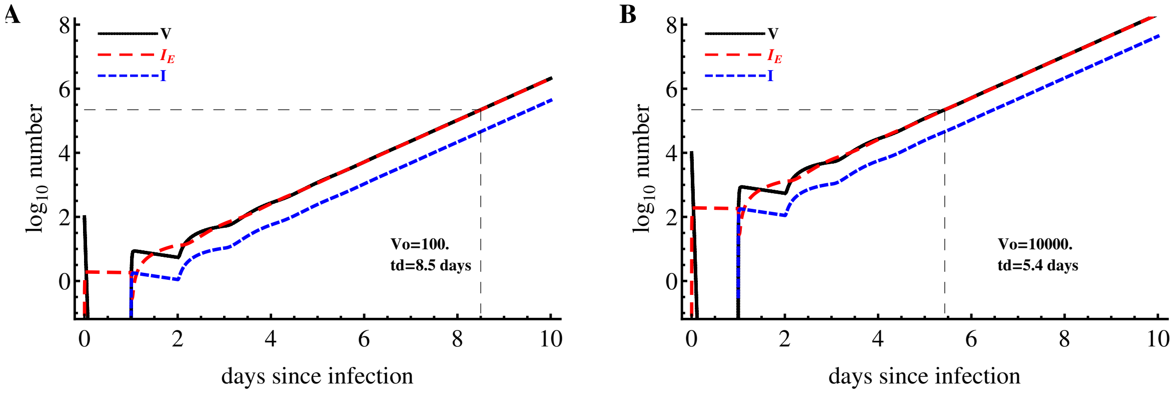

Figure S10: Dynamics of cell populations in the model with an eclipse phase having a fixed duration Δ. We simulated dynamics of the virus-infected cells and cell-free virus using eqns. (S19)–(S20) using the following parameters: $N = 10^3$, $\delta_{I_E} = 0.05$ day^{-1}, $\Delta = 1$ day, $\delta_I = 0.5$ day^{-1}, $c = 100$ day^{-1}, $\beta T = 1.95$ day^{-1}, $V_0 = 100$ (panel A) or $V_0 = 10^4$ (panel B). Lines denote the predicted number of viruses (solid black line), cells in the eclipse phase (large dashings red line), and virus-producing cells (small dashings blue line). The time to virus detection is denoted as t_d.

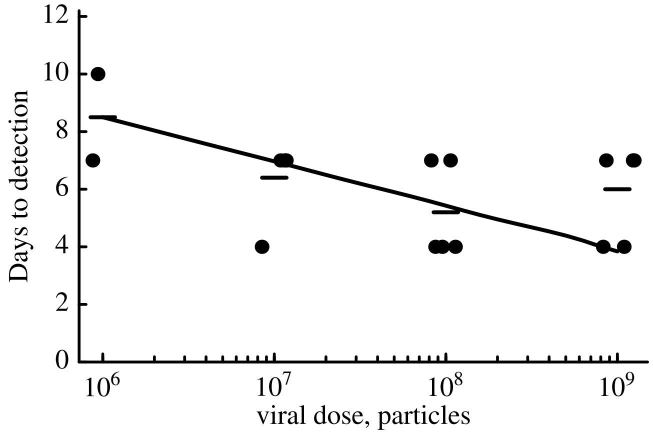

Figure S11: The model with an eclipse phase having a fixed duration does not accurately describe the decline in the time to virus detection with increasing viral doses. We simulated the virus dynamics using eqns. (S19)–(S20) using parameters shown in Figure S11 with V_0 varied from 10^2 infectious viruses (corresponding to the 10^6 total viruses) to 10^5 infectious viruses. The limit of detection in these simulations was set to $V_{\rm det} = 2.2 \times 10^5$. The time of virus detection is shown by the solid line. Dots denote experimentally measured times to virus detection from Liu *et al.* [16] and horizontal lines are the average times per given dose.

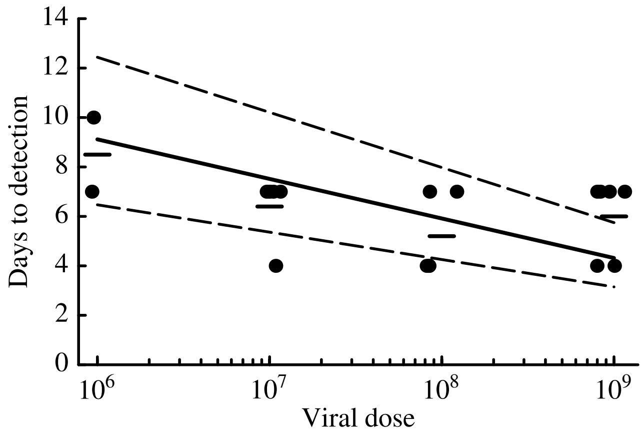

Figure S12: Simple mathematical model for SIV dynamics fails to accurately predict change in time to virus detection with increasing doses when there is variability in the rate of virus replication. We vary the virus replication rate r in accord with lognormal distribution (more precisely, normal distribution of log-transformed values for r with parameters $\overline{\log_{10} r} = 0.18$ ($\bar{r} = 1.5$ day^{-1}) and $\sigma_{\log_{10} r} = 0.07$) and run deterministic simulations in the basic mathematical model (eqns. (1)-(3)). We vary the initial number of viruses and calculate the time when viruses reach V_{det} level. The average time is shown by the solid line and thin dashed lines are the observed 95% confidence intervals from the simulations. Dots denote experimentally measured times to virus detection from Liu *et al.* [16] and horizontal lines are the average times per given dose. Parameters used in simulations are $N = 10^3$, $\delta_{I_E} = 0.05$ day^{-1}, $m = 1$ day^{-1}, $\delta = 1$ day^{-1}, $c = 100$ day^{-1}, $p_{\mathrm{inf}} = 0.33$, $V_{\mathrm{det}} = 5 \times 10^5$. In simulations we assume that the initial dose of 10^6 viral particles leads to an infection with the initial $V_0 = 88$ viruses.
